# Death-associated protein kinase 2 (DAPK2) propagates endoplasmic reticulum stress in macrophages to worsen sepsis through HSPA5-IRE1α axis

**DOI:** 10.1186/s43556-026-00501-9

**Published:** 2026-06-23

**Authors:** Yin Ni, Guo-Zhen Tang, Chen Qiu, Ge Zhu, Shu-Wen Jin, Hai-Ping Zhu, Shi-Jing Mo, Xiang-Ming Fang

**Affiliations:** 1 Department of Anesthesiology and Intensive Care, School ofMedicine, The First Affiliated Hospital, Zhejiang University, Hangzhou, 310003 People’s Republic of China; 2 Emergency and Intensive Care Unit Center, Intensive Care Unit, Zhejiang, Provincial People’s Hospital (Affiliated People’s Hospital), Hangzhou Medical College, Hangzhou, 310014 Zhejiang People’s Republic of China; 3 Center for Rehabilitation Medicine, Department of Intensive Rehabilitation Care, Unit, Zhejiang Provincial People’s Hospital (Affiliated People’s Hospital), Hangzhou, Medical College, Hangzhou, 310014 Zhejiang People’s Republic of China; 4 Center for Rehabilitation Medicine, Rehabilitation & Sports Medicine Research, Institute of Zhejiang Province, Department of Rehabilitation Medicine, , Zhejiang, Provincial People’s Hospital (Affiliated People’s Hospital), Hangzhou Medical, College, Hangzhou, 310014 Zhejiang People’s Republic of China; 5 Department of Ultrasound in Medicine, Second Affiliated Hospital of Zhejiang University School of Medicine, Hangzhou, 310003 Zhejiang People’s Republic of China; 6 Center for Veterinary Sciences, Zhejiang University, Hangzhou, 310058 Zhejiang People’s Republic of China; 7 Zhejiang Lab, Hangzhou, 311121 Zhejiang People’s Republic of China; 8 Department of Intensive Care Unit, The First Affiliated Hospital, Wenzhou Medical University, Wenzhou, 325000 Zhejiang People’s Republic of China

**Keywords:** DAPK2, HSPA5, IRE1α, Sepsis

## Abstract

Sepsis is associated with a pronounced but poorly understood endoplasmic reticulum stress (ERS) response. In this study, we found that death-associated protein kinase 2 (DAPK2), a calcium/calmodulin-regulated serine/threonine kinase, exhibits elevated expression in macrophages from patients with sepsis and from septic mice. Macrophage DAPK2 expression is transcriptionally upregulated through the activation of the Toll-like receptor 4 (TLR4)–myeloid differentiation primary response 88 (MyD88)–nuclear factor-κB (NF-κB) pathway. Macrophage-specific deletion of DAPK2 attenuated sepsis severity and mitigated the ERS response. Using liquid chromatography–tandem mass spectrometry (LC–MS/MS), we identified heat shock protein family A member 5 (HSPA5) as a binding partner for DAPK2. Because DAPK2 function had been previously associated with the kinase activity, we speculated that it might control ERS of macrophages through HSPA5 phosphorylation. Further investigation indeed revealed that DAPK2 phosphorylates HSPA5 at serine-588, which promotes the proteasomal degradation of HSPA5 and subsequently leads to the activation of inositol-requiring enzyme 1α (IRE1α). Inhibition of HSPA5 exacerbated sepsis in mice with macrophage-specific DAPK2 deficiency; however, this effect was abrogated by the deactivation of IRE1α. In conclusion, our findings demonstrate that DAPK2 propagates macrophage ERS through the HSPA5–IRE1α axis during systemic infection, suggesting this pathway as a potential therapeutic target in sepsis.

**Supplementary Information:**

The online version contains supplementary material available at 10.1186/s43556-026-00501-9.

## Introduction

Sepsis, a life-threatening organ dysfunction caused by a dysregulated host response to infection, remains a leading cause of morbidity and mortality among critically ill patients worldwide [1]. The host defense against pathogenic invasion is initiated by the recognition of pathogen-associated molecular patterns (PAMPs) by pattern recognition receptors (PRRs), a process accompanied by the recruitment of innate immune cells and the production of inflammatory mediators [2, 3]. Although macrophage dysfunction is considered a key factor in the severity of infection, the specific molecular components governing immune homeostasis in sepsis remain incompletely understood. Elucidating these mechanisms is essential for the development of novel therapeutic strategies for this devastating disease.

As the most plastic innate immune cells in maintaining tissue homeostasis, macrophages have been extensively characterized in the context of a response to pathogenic invasion and their dysfunctions have been considered to represent the extent of infection dissemination. To date, the detailed mechanisms wherein macrophages regulate immunity of sepsis has not been scrutinized. There is an urgent scientific need to uncover novel molecular components responsible for immunomodulation and elucidate their mechanisms of function in sepsis, which may have valuable scientific implications and would be helpful to guide therapy and improve clinical care of septic illness.

Cellular responses to various stresses during infection has attracted increased attention and might constitute an etiology for progression of systemic inflammation. The death-associated protein kinase (DAPK) family comprises calmodulin-regulated serine/threonine kinases that are implicated in diverse cellular processes, including T cell trafficking [4], anoikis [5], cell cycle arrest [6] and necroptosis [7, 8]. DAPK activation is critically involved in a range of physiological and pathological conditions, such as erythroblast formation [9], immune surveillance [10], vascular calcification [11], hypertension [12], Alzheimer's disease [13] and diabetic cardiomyopathy [14]. On the other hand, the capacity of DAPK to instruct inflammation is just beginning to be uncovered. Among the three highly conserved mammalian isoforms, DAPK2 shares an N-terminal catalytic domain with 80% homology to the DAPK1 kinase domain [15]. Although our recent research has established an essential role for DAPK1 in redox inflammation [16], the precise function and molecular mechanisms of DAPK2 in sepsis remain enigmatic.

Recent investigations into the pathogenesis of inflammatory disorders have revealed the integral involvement of endoplasmic reticulum stress (ERS) [17–20]. A major contributor to the initiation of ERS in immune cells is the activation of inositol-requiring enzyme 1α (IRE1α), an evolutionarily conserved ER transmembrane protein [21, 22]. Under physiological conditions, IRE1α is sequestered in an inactive monomeric state by heat shock protein family A member 5 (HSPA5), also known as glucose-regulated protein 78 (GRP78) or BiP [23]. HSPA5 has emerged as a potential prognostic biomarker in septic patients, suggesting its clinical relevance in microbial infections [24]. However, the role of the HSPA5–IRE1α axis in the context of sepsis has not been thoroughly investigated.

In this study, we combined in silico, biochemical, cell and animal studies to demonstrate that DAPK2 triggers the phosphorylation and subsequent proteasomal degradation of HSPA5, leading to IRE1α activation, thereby propagating ERS in macrophages and exacerbating sepsis. Our findings gain novel insights into the molecular mechanisms underlying systemic inflammation, which would be helpful to provide viable therapeutic strategies for sepsis.

## Results

### DAPK2 is Highly Expressed in Macrophages of Patients with Sepsis and in Septic Mice

**Fig. 1:**
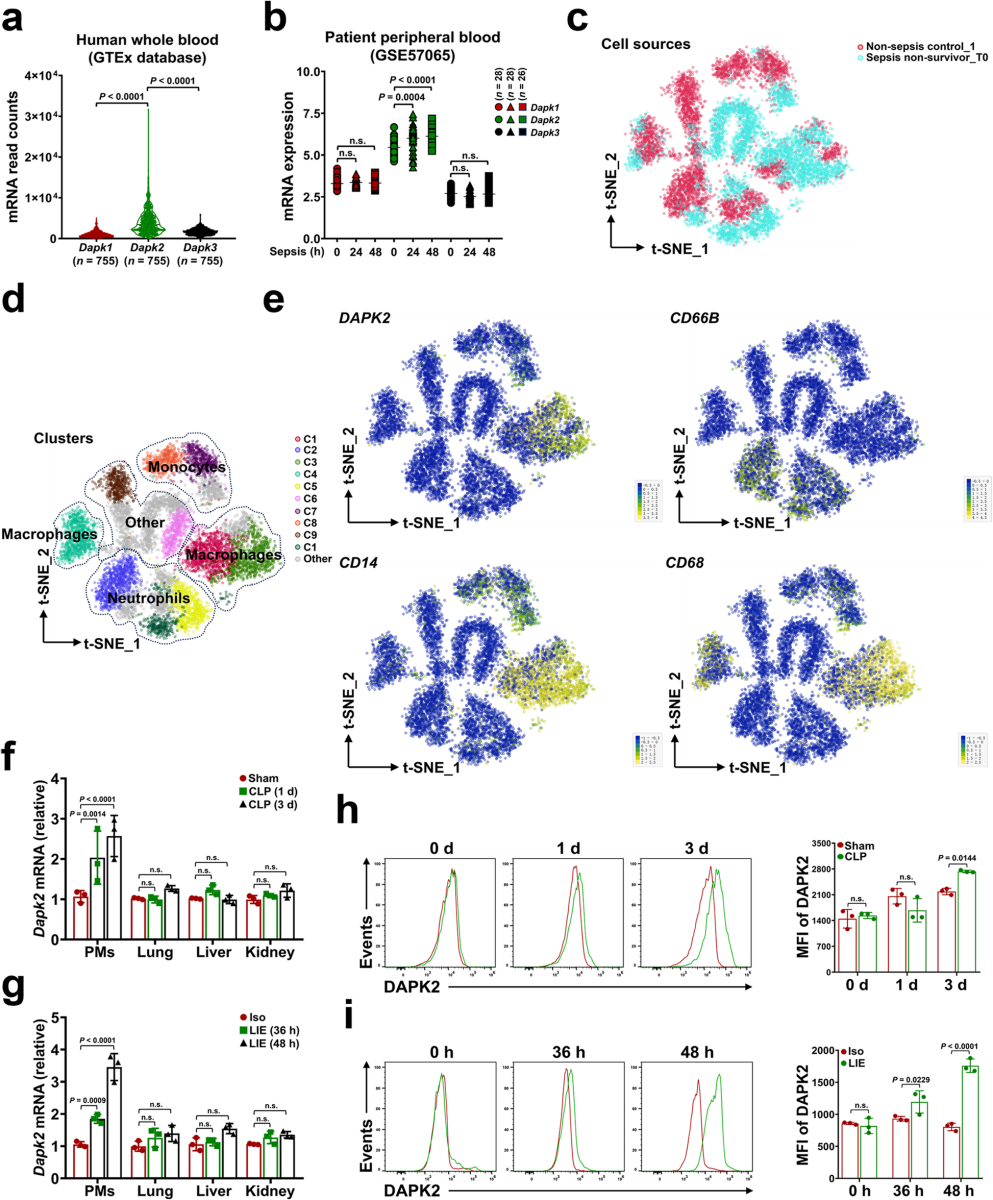
DAPK2 is transcriptionally upregulated in macrophages of septic patients and mice. **a** Levels of DAPK1-DAPK3 mRNAs in human blood using the Genotype-Tissue Expression (GTEx) database (*n* = 755 per group). **b** The mRNA expression of DAPK1-DAPK3 genes in the publically available dataset (GSE57065) from the NCBI GEO (*n* = 28 or 26 per group). **c** Cell sources of sc-RNA-seq data from GSE167363 in which sample 1 from non-sepsis control and sample T0 of sepsis non-survivors were included for analysis. **d** tSNE plot of cell clusters of scRNA-seq data in c. **e** tSNE plots of DAPK2, CD68, CD14 and CD66B of scRNA-seq data in d. **f** RT-qPCR comparing DAPK2 mRNA expression in peritoneal macrophages (PMs), lung, liver and kidney tissues of mice with cecal ligation and puncture (CLP) operation (*n* = 3 per group). d, day; n.s., no significant. **g** RT-qPCR comparing DAPK2 mRNA expression in peritoneal macrophages (PMs), lung, liver and kidney tissues of mice with LPS-induced endotoxemia (LIE) challenge (*n* = 3 per group). h, hour; Iso, isotype; n.s., no significant. **h** Histogram plots and quantification of flow cytometry for measurement of intracellular DAPK2 staining in peritoneal macrophages (PMs) from mice with cecal ligation and puncture (CLP) operation at the indicated times (*n* = 3 per group). d, day; n.s., no significant. **i** Histogram plots and quantification of flow cytometry for measurement of intracellular DAPK2 staining in peritoneal macrophages (PMs) from mice following LPS-induced endotoxemia (LIE) challenge at the indicated times (*n* = 3 per group). h, hour; Iso, isotype; n.s., not significant. Data are expressed as mean ± s.d. (a, b and f-i). Two-sided ANOVA with Bonferroni post hoc *t* test correction (a, b and f-i) was used to calculate the *P* value, respectively

To investigate the potential role of DAPK-family kinases in sepsis, we first evaluated the basal transcript levels of DAPK1, DAPK2, and DAPK3 in human whole blood using the Genotype-Tissue Expression (GTEx) database. This analysis indicated that DAPK2 had the highest basal mRNA read counts among the three DAPK homologues (Fig. 1a). We next examined publicly available transcriptomic data from the Gene Expression Omnibus (GEO) [25] and found that DAPK2 mRNA expression, but not that of DAPK1 or DAPK3, was significantly increased in peripheral blood samples from patients with sepsis (Fig. 1b). To further define the cellular source of this upregulation, we analyzed a single-cell RNA-sequencing (scRNA-seq) dataset of blood samples from septic patients and non-septic controls [26]. This analysis revealed that DAPK2 was predominantly upregulated in the macrophage and monocyte cluster relative to other cell populations (Fig. 1c-e and Fig. S1).

To determine whether DAPK2 expression is similarly regulated in experimental models of sepsis, we quantified DAPK2 mRNA in peritoneal macrophages (PMs) from mice subjected to polymicrobial sepsis induced by cecal ligation and puncture (CLP). Although DAPK2 mRNA levels in other tissues were only marginally affected, its expression was progressively upregulated in PMs after the CLP procedure (Fig. 1f). Similar results were observed in mice with LPS-induced endotoxemia induced by intraperitoneal (i.p.) injection of lipopolysaccharide (LPS) (termed as LIE hereafter) (Fig. 1g). Notably and in agreement with these results, DAPK2 protein levels were also markedly increased in PMs from mice after CLP or LPS challenge (Fig. 1h and i).

### DAPK2 Upregulation in Macrophages is Mediated by TLR4–MyD88–NF-κB Signaling

**Fig. 2:**
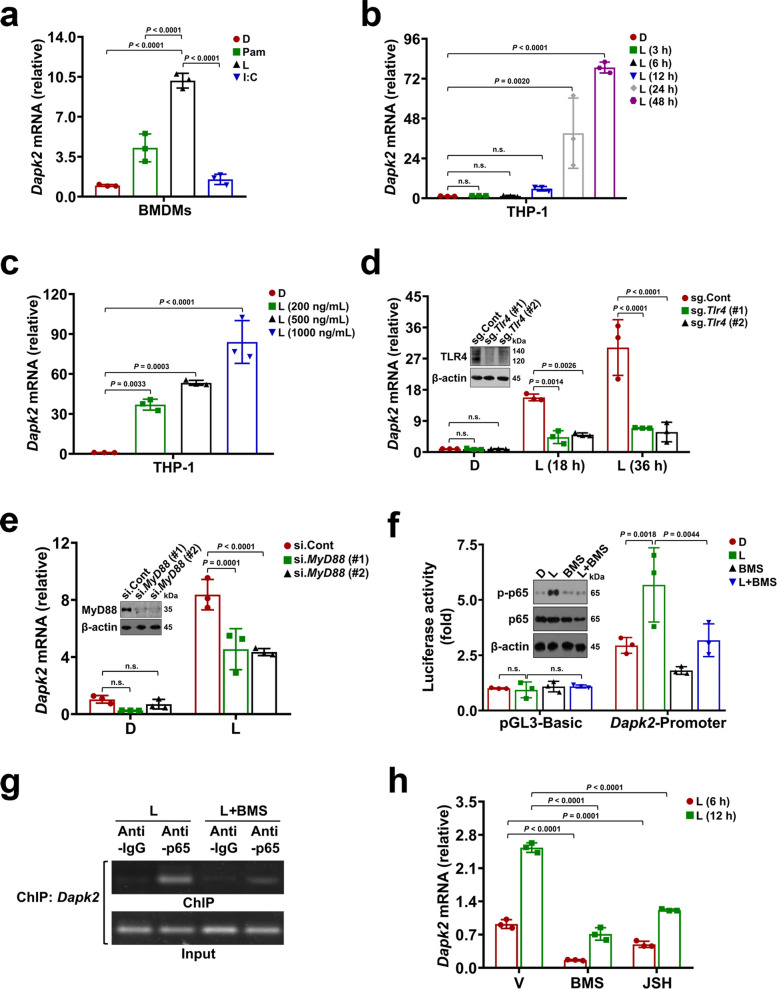
DAPK2 is transcriptionally upregulated in macrophages by TLR4/MyD88-mediated NF-κB activation. **a** RT-qPCR evaluating DAPK2 mRNA expression in primary murine bone marrow-derived macrophages (BMDMs) stimulated with DMSO (D), LPS (L), Pam3CSK4 (Pam) or poly(I:C) (I:C) for 24 h (*n* = 3 per group). DMSO, dimethyl sulfoxide. **b** RT-qPCR determining DAPK2 mRNA expression in human THP-1 macrophages stimulated with LPS (L) at the indicated times (*n* = 3 per group). D, DMSO. **c** RT-qPCR measuring DAPK2 mRNA expression in human THP-1 macrophages stimulated with LPS (L) for 24 h at the indicated concentrations (*n* = 3). D, DMSO. **d** RT-qPCR comparing DAPK2 mRNA expression in THP-1 macrophages stimulated with LPS (L) at the indicated times upon transfection with small guide RNA (sgRNA) targeting control (sg.Cont) or *Tlr4* (sg.*Tlr4*) (*n* = 3 per group). *Inset:* Immunoblotting of TLR4 protein levels in THP-1 macrophages transfected with sg.RNA targeting control (sg.Cont) or *Tlr4* (sg.*Tlr4*). D, DMSO. **e** RT-qPCR assessing DAPK2 mRNA expression in the LPS-stimulated human THP-1 macrophages with small interfering RNA (siRNA) targeting control (si.Cont) or MyD88 (si.*MyD88*) transfection (*n* = 3 per group). *Inset:* Immunoblotting of MyD88 protein levels in human THP-1 macrophages transfected with siRNA targeting control (si.Cont) or MyD88 (si.*MyD88*). D, DMSO. **f** Luciferase assays of DAPK2 promoter activity in human THP-1 macrophages with LPS (L) plus BMS345541 (BMS) cotreatment versus LPS single treatment (*n* = 3 per group). *Inset:* Immunoblotting of p65 phosphorylation in human THP-1 macrophages with the indicated treatment. D, DMSO. **g** ChIP assay detecting p65 binding to the promoter of DAPK2 gene. L, LPS; BMS, BMS345541. **h** RT-qPCR evaluating DAPK2 mRNA expression in the BMS345541 (BMS)- or JSH-23 (JSH)-pretreated human THP-1 macrophages in the presence of LPS stimuli for the indicated times (*n* = 3 per group). V, vehicle. Data are expressed as mean ± s.d. (a-f and h). Two-sided ANOVA with Bonferroni post hoc *t* test correction (a-f and h) was used to calculate the *P* value

Given activation of Toll-like receptor 4 (TLR4) initiates a myeloid differentiation primary response 88 (MyD88)–dependent nuclear factor-κB (NF-κB) signaling cascade that drives inflammation [27], we investigated whether this pathway governs the transcriptional upregulation of DAPK2. Stimulation of primary murine bone marrow-derived macrophages (BMDMs) with the TLR4 agonist lipopolysaccharide (LPS) resulted in a more potent induction of DAPK2 mRNA than did stimulation with agonists for TLR1/2 (Pam3CSK4) or TLR3 (poly[I:C]) (Fig. 2a). A time- and dose-dependent increase in DAPK2 expression was also observed in human THP-1 macrophages after LPS stimulation (Fig. 2b and c). This induction was dependent on TLR4, as it was absent in TLR4-deficient macrophages (Fig. 2d) and on the adaptor protein MyD88, as siRNA-mediated silencing of MyD88 markedly attenuated the LPS-induced expression of DAPK2 (Fig. 2e). Analysis of the nucleotide sequence of DAPK2 gene promoter region using JASPAR database [28] identified a functional NF-κB binding element AGGGCTTTCC resided in −1088 bp to −1079 bp range, which was similar to the NF-κB binding consensus element GGGRNNYYCC (N, any base; R, purine; and Y, pyrimidine) [29]. To assess the functional relevance of this site, we constructed a DAPK2 promoter luciferase reporter. LPS stimulation significantly increased reporter activity, an effect that was abrogated by the NF-κB inhibitor BMS345541 (Fig. 2f). Chromatin immunoprecipitation (ChIP) assays confirmed the LPS-inducible binding of NF-κB subunit p65 to DAPK2 in the absence of BMS345541 treatment (Fig. 2g). Furthermore, inhibiting the nuclear translocation of p65 with JSH-23 also prevented the LPS-induced upregulation of DAPK2 (Fig. 2h). Collectively, these data demonstrate that DAPK2 is transcriptionally upregulated in macrophages through the TLR4–MyD88–NF-κB pathway in response to inflammatory stimuli.

### Macrophage DAPK2 exacerbates sepsis-associated lethality, cytokine storm, and cell death

**Fig. 3:**
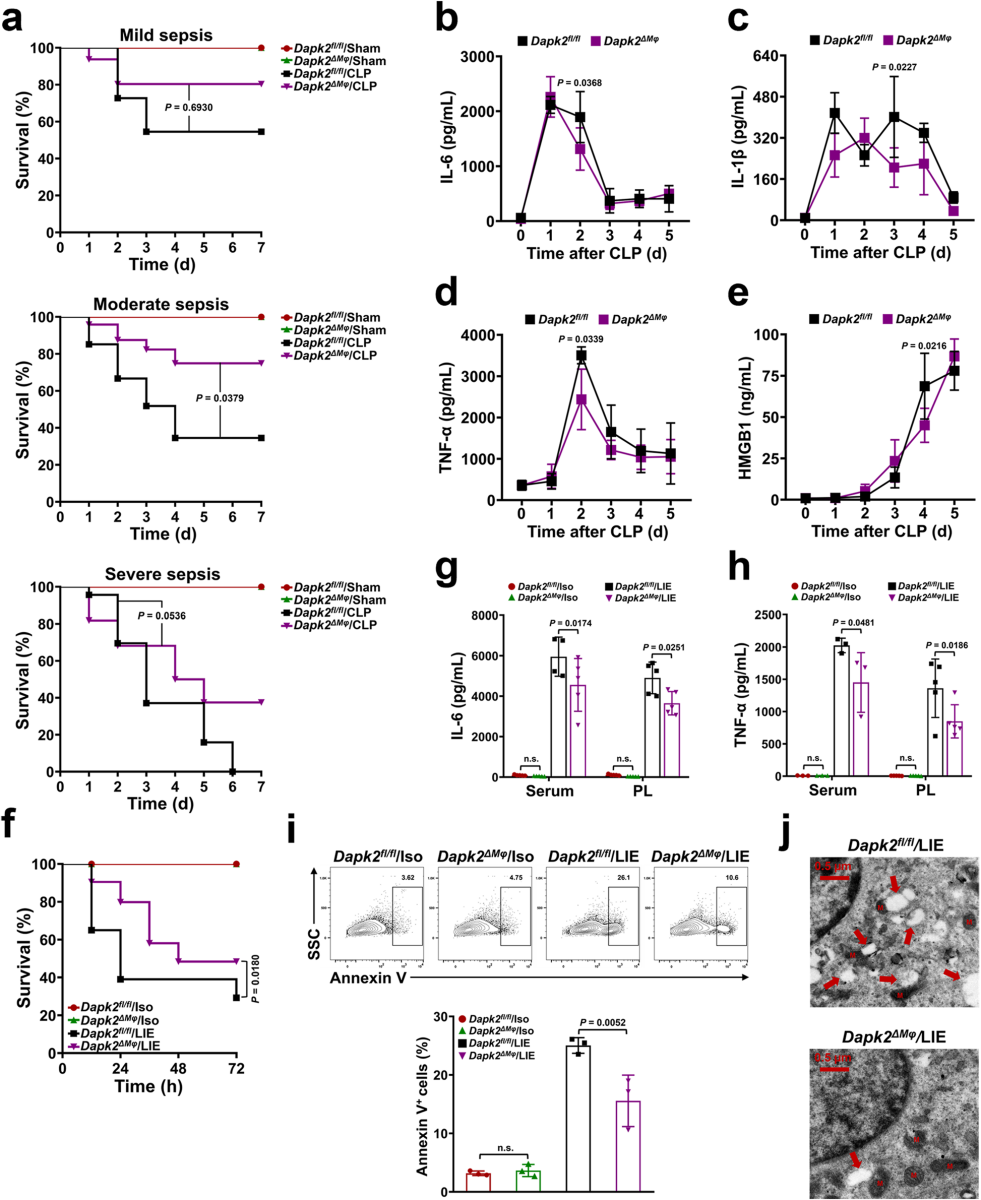
Macrophage DAPK2 deficiency endows mice refractory to sepsis and prohibits ERS of macrophages. **a** Kaplan–Meier curves analyzing survivals of *Dapk2*^*fl/fl*^ versus *Dapk2*^*ΔMφ*^ mice after cecal ligation and puncture (CLP) operation with syringe needles with gauges ranging from 27G (‘‘Mild sepsis’’), 22G (‘‘Moderate sepsis’’) to 17G (‘‘Severe sepsis’’) at the indicated times (*n* ≥ 15 per group). d, day. **b-e** Enzyme-linked immunosorbent assay (ELISA) assays testing levels of interleukin-6 (IL-6, b), interleukin-1β (IL-1β, c), tumor necrosis factor-α (TNF-α, d) and high mobility group box 1 (HMGB1, e) in serum of *Dapk2*^*ΔMφ*^ versus *Dapk2*^*fl/fl*^ mice after cecal ligation and puncture (CLP) operation at the indicated times (*n* = 3 per group). **f** Kaplan–Meier curves analyzing survivals of *Dapk2*^*ΔMφ*^ versus *Dapk2*^*fl/fl*^ mice at the indicated times following LPS-induced endotoxemia (LIE) challenge (*n* ≥ 15 per group). h, hour. **g, h** Enzyme-linked immunosorbent assay (ELISA) assays comparing levels of interleukin-6 (IL-6, g) and tumor necrosis factor-α (TNF-α, h) in serum and peritoneal lavage (PL) fluid of *Dapk2*^*ΔMφ*^ versus *Dapk2*^*fl/fl*^ mice following LPS-induced endotoxemia (LIE) challenge (*n* ≥ 3 per group). n.s., no significant. **i** Contour plots and quantification of flow cytometry with Annexin V staining in PMs of *Dapk2*^*ΔMφ*^ versus *Dapk2*^*fl/fl*^ mice following LPS-induced endotoxemia (LIE) challenge (*n* = 3 per group). Iso, isotype control; n.s., no significant. **j** Transmission electron microscope (TEM) images of PMs from *Dapk2*^*ΔMφ*^ and *Dapk2*^*fl/fl*^ mice with LPS-induced endotoxemia (LIE) challenge. Significant ER damage was marked by red arrows. M, mitochondria. Data are expressed as mean ± s.d. (b-e and g-i). Log-rank t test (a and f) and Two-sided ANOVA with Bonferroni post hoc *t* test correction (b-e and g-i) was used to calculate the *P* value, respectively

The transcriptional upregulation of DAPK2 in macrophages under inflammatory circumstances prompted us to dissect its functional role in sepsis. To this end, we adopted *Lyz2-Cre;Dapk2*^*fl/fl*^ (*Dapk2*^*ΔMφ*^) mice in which both mRNA and protein expression of DAPK2 were abolished in BMDMs as compared with the *Dapk2*^*fl/fl*^ littermates (Fig. S2a and b). In models of moderate and severe sepsis induced by CLP, *Dapk2*^*ΔMφ*^ mice exhibited significantly increased resistance to mortality in comparison to *Dapk2*^*fl/fl*^ mice (Fig. 3a). Consistent with this finding, CLP-operated *Dapk2*^*ΔMφ*^ mice displayed an attenuated cytokine storm relative to their *Dapk2*^*fl/fl*^ counterparts (Fig. 3b-e). However, no significant differences were observed in the bacterial burden at distinct infectious sites or in bacterial translocation into the blood between the two genotypes (Fig. S2c and d). This suggests that the protective effect of DAPK2 deficiency is not primarily attributable to an enhanced bactericidal capacity of macrophages. Following LIE challenge, *Dapk2*^*ΔMφ*^ mice showed a significantly higher survival rate (Fig. 3f), which was accompanied by decreased production of inflammatory cytokines in the serum and peritoneal lavage fluid (Fig. 3g and h) and alleviated lung and liver injuries (Fig. S2e-g). Programmed cell death of macrophages is a known contributor to immunosuppression in sepsis [30]. Flow cytometric analysis of PMs revealed a reduced percentage of annexin V-positive cells in *Dapk2*^*ΔMφ*^ mice after both CLP and LIE challenges as compared with their respective *Dapk2*^*fl/fl*^ controls (Fig. 3i and S2h). Considering the significance of programmed cell death in sepsis, we elucidated the type of macrophage death that engages in DAPK2ʼs role. Either CLP operation or LIE challenge led to prominent caspase-3 cleavage, an indicator of apoptosis, in PMs of *Dapk2*^*fl/fl*^ mice but was unable to do so in those of *Dapk2*^*ΔMφ*^ mice (Fig. S2i and j), demonstrating that loss of *Dapk2* inhibits apoptosis of macrophages during sepsis. To clarify whether the observed cell death could be occurred in the form of pyroptosis, we stained PMs with FAM-YVAD-FMK (FLICA), a cell-permeable probe that binds to active caspase-1, and gasdermin D (GSDMD) p30, respectively. We found that *Dapk2*^*ΔMφ*^ mice with CLP operation or LIE challenge had similar amounts of FLICA and GSDMD p30 to *Dapk2*^*fl/fl*^ mice with the same operation/challenge (Fig. S2i and j). We then asked whether DAPK2 is required for necroptosis in which mixed lineage kinase domain-like protein (MLKL) undergoes phosphorylation. Phosphorylation of MLKL was hardly detectable in both genotypes of the control groups. Upon CLP operation and LIE challenge, however, levels of MLKL phosphorylation were pronouncedly enhanced in *Dapk2*^*fl/fl*^ mice, while such phenomenon was deterred in *Dapk2*^*ΔMφ*^ mice (Fig. S2i and j).

DAPK2 has been reported to be an inhibitor of mammalian target of rapamycin 1 (mTORC1) [31]. Treatment with the mTORC1 inhibitor rapamycin did not significantly affect the survival advantage of *Dapk2*^*ΔMφ*^ mice in either the CLP or LIE models (Fig. S3a and b), suggesting that DAPK2 exacerbates sepsis through a mechanism independent of mTORC1 inhibition. The transition of macrophage from a pro-inflammatory M1 phenotype toward an anti-inflammatory M2 subtype confers inflammation resolution [32]. We examined the role of DAPK2 in macrophage polarization. The proportions of pro-inflammatory M1-like (CD11c⁺F4/80⁺) and anti-inflammatory M2-like (CD206⁺F4/80⁺) macrophages did not differ significantly between *Dapk2*^*fl/fl*^ and *Dapk2*^*ΔMφ*^ BMDMs after stimulation with lipopolysaccharide (LPS) plus interferon-γ (IFN-γ) or with interleukin-4 (IL-4), respectively (Fig. S3c and d). These results indicate that DAPK2 does not substantially influence macrophage phenotypic transition in this context. Collectively, these data strongly suggest that DAPK2 in macrophages worsens septic lethality, exacerbates the cytokine storm and promotes macrophage death.

### DAPK2 directly interacts with HSPA5

**Fig. 4:**
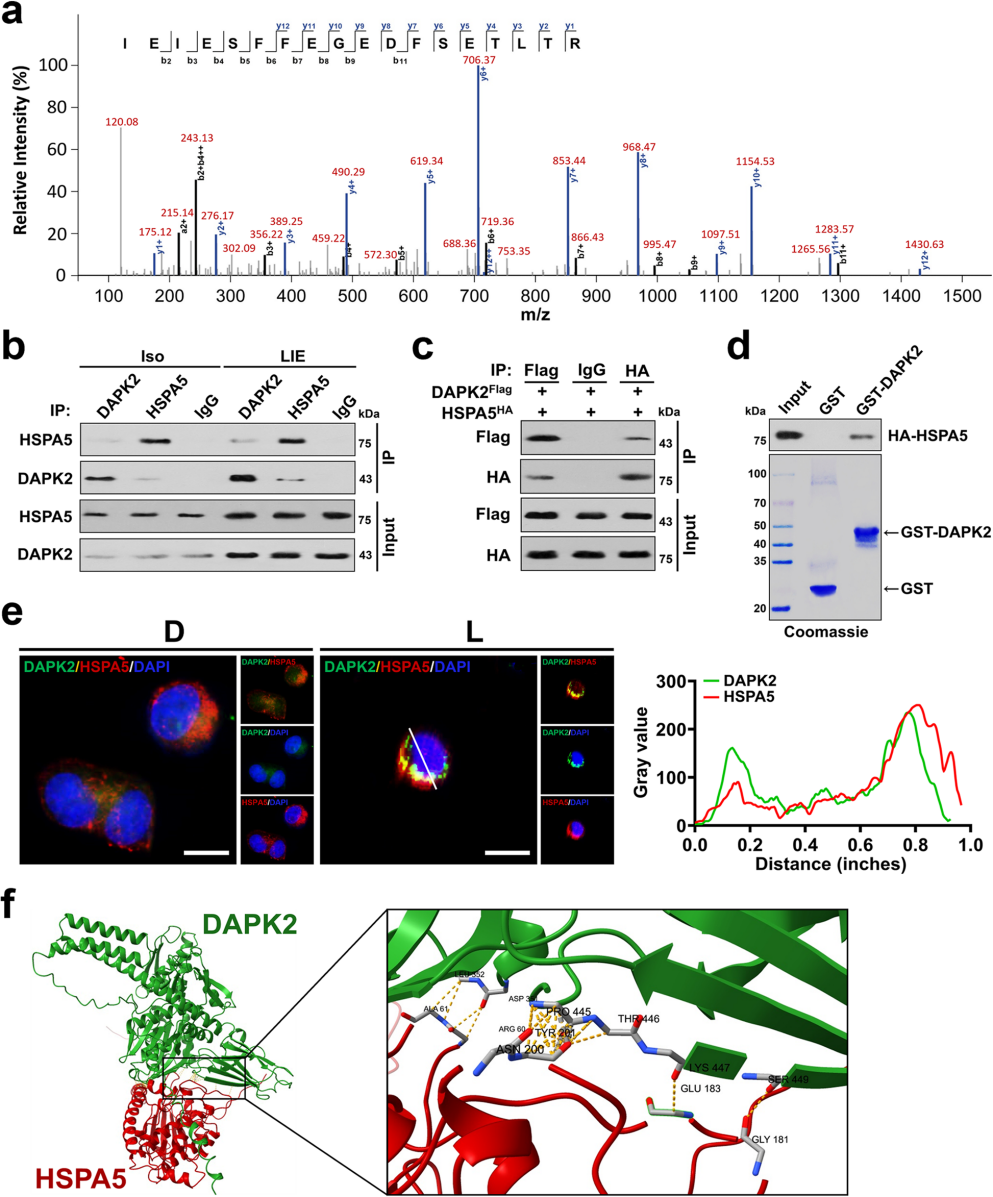
DAPK2 directly interacts with HSPA5. **a** LC–MS/MS spectrum corresponding to ^308^IEIESFFEGEDFSETLTR^325^ from HSPA5. The indicated b- and y-ion series are marked. **b** Coimmunoprecipitation assay examining the interaction of DAPK2 with HSPA5 in PMs from mice following LPS-induced endotoxemia (LIE) challenge. **c** Coimmunoprecipitation assay detecting the interaction between DAPK2 and HSPA5 in HEK293T cells transfected with Flag-tagged wild-type DAPK2 (DAPK2^Flag^) and HA-tagged wild-type HSPA5 (HSPA5^HA^). **d** GST-pulldown assay with mixing purified GST-DAPK2 and IPs of HA-tagged wild-type HSPA5 from HEK293T cells followed by immunoblotting analyses with an anti-HA antibody. **e** Representative dichromatic immunfluorescence images determining colocalization of DAPK2 and HSPA5 in primary BMDMs with LPS stimuli. D, DSMO; L, LPS. Scale bars: 10 μm. **f** Structural model of the HSPA5-DAPK2 complex predicted by AlphaFold-Multimer

Organelles with diverse morphological alterations upon stress are major sensors of damage and death signaling pathways integrated by plasma-membrane receptors [33]. Transmission electron microscope (TEM) analyses of PMs from LIE-challenged *Dapk2*^*fl/fl*^ and *Dapk2*^*ΔMφ*^ mice showed that DAPK2 deficiency prohibited endoplasmic reticulum (ER) dilation with ribosome shedding (Fig. 3j), an indicative of ER stress (ERS), the cellular stress response that results from an accumulation of unfolded or misfolded proteins in the endoplasmic reticulum. To identify the downstream effector of DAPK2 responsible for macrophage ERS, we performed pulldown assays with purified His-tagged DAPK2 from lysates of LIE-challenged *Dapk2*^*ΔMφ*^ PMs, followed by identification with liquid chromatography with tandem mass spectrometry (LC–MS/MS). Through this approach, HSPA5 was identified as a DAPK2-interacting protein with representative MS spectrum corresponding to ^308^IEIESFFEGEDFSETLTR^325^ (Fig. 4a). This interaction was validated through several methods. Reciprocal co-immunoprecipitation (co-IP) assays with antibodies against DAPK2 and HSPA5 from lysates of LIE-challenged macrophages confirmed an endogenous interaction (Fig. 4b). In human embryonic kidney 293 T (HEK293T) cells co-transfected with constructs for hemagglutinin (HA)-tagged HSPA5 and Flag-tagged DAPK2, co-IP assays also showed a clear interaction (Fig. 4c). Furthermore, an in vitro glutathione S-transferase (GST) pulldown assay using purified GST-DAPK2 and immunoprecipitated HA-HSPA5 validated a direct interaction between the two proteins (Fig. 4d). Confocal microscopy revealed that HSPA5 and DAPK2 showed increased colocalization in response to LPS stimulation (Fig. 4e). To gain structural insight, we generated a model of the DAPK2–HSPA5 complex using AlphaFold-Multimer [34], which predicted that the positively charged residue R60 of HSPA5 packs against the negatively charged residue D351 of DAPK2, and the negatively charged residue E183 of HSPA5 packs against the positively charged residue K447 of DAPK2. Moreover, A61, N200, Y201 and G181 of HSPA5 and L352, P445, T446 and S449 of DAPK2 were predicted to contribute to the hydrophobic interactions (Fig. 4f). The interaction was independent of DAPK2 kinase activity, as a kinase-dead K52A DAPK2 mutant could still be co-immunoprecipitated with HSPA5 (Fig. S4a). Together, these results indicate that DAPK2 directly interacts with HSPA5.

### DAPK2 Phosphorylates HSPA5 at Serine 588, Promoting its Proteasomal Degradation

**Fig. 5:**
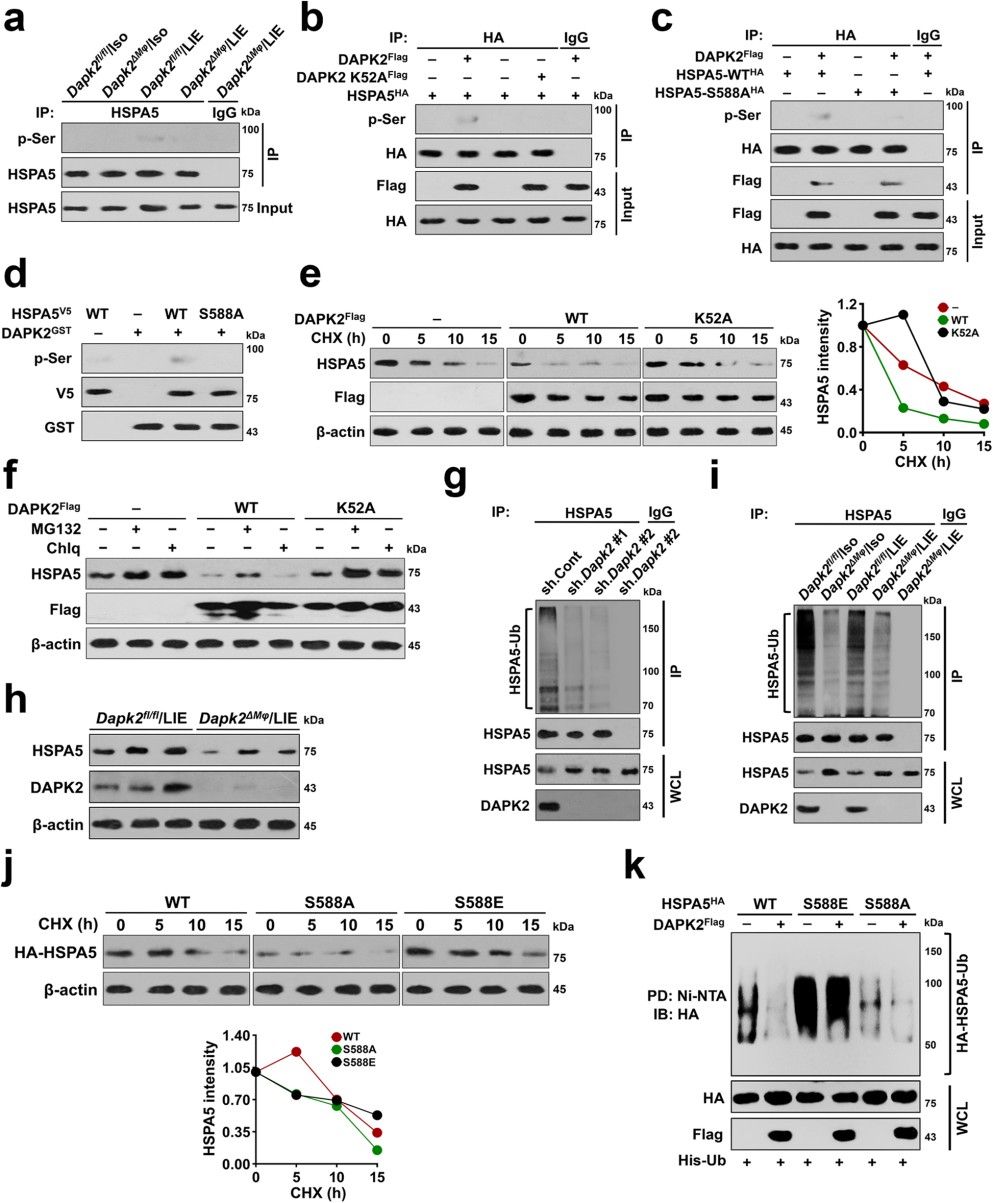
DAPK2 phosphorylates HSPA5 at Ser588 to regulate HSPA5 stabilization. **a** Coimmunoprecipitation assay assessing HSPA5 Ser phosphorylation in PMs from *Dapk2*^*fl/fl*^ and *Dapk2*^*ΔMφ*^ mice with LIE challenge. **b** Coimmunoprecipitation assay comparing HSPA5 Ser phosphorylation in HEK293T cells cotransfected with Flag-tagged wild-type DAPK2 (DAPK2^Flag^) or Flag-tagged K52A mutant DAPK2 (DAPK2 K52A^Flag^) and HA-tagged wild-type HSPA5. **c** Coimmunoprecipitation assay determining HSPA5 Ser phosphorylation in HA-tagged wild-type HSPA5 (HSPA5-WT^HA^)- and mutant HSPA5 Ser588A (HSPA5-S588A^HA^)-expressed HEK293T cells with or without Flag-tagged wild-type DAPK2 (DAPK2^Flag^) transfection. **d** In vitro kinase assay with incubating active DAPK2 protein with purified V5-tagged wild-type HSPA5 or S588A mutant HSPA5 followed by immunoblotting analyses with an anti-pSer antibody. **e** Cycloheximide (CHX) pulse-chase experiments comparing the turnover of HSPA5 protein in HEK293T cells transfected with Flag-tagged wild-type DAPK2 (WT) or Flag-tagged K52A mutant DAPK2 (K52A). **f** Immunoblotting analyses testing abundance of HSPA5 protein in HEK293T cells with Flag-tagged wild-type DAPK2 (WT) or Flag-tagged K52A mutant DAPK2 (K52A) transfection in the presence or absence of MG132 and chloroquine (Chlq) treatment. **g** Cellular ubiquitination assays comparing polyubiquitylation levels of HSPA5 in HEK293T cells with DAPK2 shRNA (sh.*Dapk2*) transfection. WCL, whole cell lysate. **h** Immunoblotting analyses evaluating amounts of HSPA5 protein in PMs from *Dapk2*^*fl/fl*^ and *Dapk2*^*ΔMφ*^ mice with LIE challenge. **i** Cellular ubiquitination assays examining polyubiquitylation levels of HSPA5 in PMs from *Dapk2*^*fl/fl*^ and *Dapk2*^*ΔMφ*^ mice with LIE challenge. **j** Cycloheximide (CHX) pulse-chase experiments assessing the turnover of HSPA5 protein in HEK293T cells with HA-tagged wild-type HSPA5 (WT), mutant HSPA5 Ser588E (S588E) and Ser588A (S588A) transfection in the presence or absence of CHX exposure for the indicated times. **k** Cellular ubiquitination assays detecting polyubiquitylation levels of HSPA5 in HEK293T cells with HA-tagged wild-type HSPA5 (WT), mutant HSPA5 Ser588E (S588E) and Ser588A (S588A) transfection. PD, pull-down; Ni–NTA, Ni^2+^-nitrilotriacetic acid

As DAPK2 is a serine/threonine kinase, we investigated whether it phosphorylates HSPA5. Immunoprecipitation of HSPA5 from PMs of LIE-challenged mice followed by immunoblotting with an anti-phospho-serine antibody revealed that HSPA5 phosphorylation was abolished in *Dapk2*^*ΔMφ*^ mice compared with *Dapk2*^*fl/fl*^ controls (Fig. 5a). In HEK293T cells, forced expression of wild-type DAPK2, but not the kinase-dead K52A mutant, induced serine phosphorylation of HSPA5 (Fig. 5b).

Analysis of the amino acid (aa) sequence of HSPA5 using GPS software (http://gps.biocuckoo.org) revealed serine 588 (Ser588) as a putative DAPK2 phosphorylation site that is evolutionarily conserved (Fig. S4b). Mutation of this residue to alanine (S588A) abolished DAPK2-induced phosphorylation of HSPA5 without affecting the DAPK2–HSPA5 interaction (Fig. 5c). An in vitro kinase assay confirmed that active DAPK2 phosphorylated wild-type HSPA5 but not the HSPA5 S588A mutant (Fig. 5d).

We next sought to determine the functional consequence of this phosphorylation on HSPA5 stability. Overexpression of wild-type DAPK2, but not the K52A mutant, decreased steady-state levels of HSPA5 protein (Fig. S4c), whereas DAPK2 depletion increased HSPA5 levels (Fig. S5d), with no corresponding changes in HSPA5 mRNA (Fig. S5e and f). Cycloheximide (CHX) pulse-chase experiments showed that DAPK2 expression accelerated HSPA5 protein turnover (Fig. 5e), while DAPK2 deficiency extended its half-life (Fig. S4g). This degradation was mediated by the ubiquitin–proteasome system (UPS), as the proteasome inhibitor MG132, but not the lysosome inhibitor chloroquine (Chlq), restored HSPA5 protein levels in DAPK2-expressing cells (Fig. 5f). Accordingly, DAPK2 depletion markedly reduced the polyubiquitylation of HSPA5 (Fig. 5g). In PMs from LIE-challenged *Dapk2*^*ΔMφ*^ mice, HSPA5 protein levels were increased (Fig. 5h) and its polyubiquitylation was decreased (Fig. 5i). To directly link phosphorylation to degradation, we used HSPA5 mutants. A phospho-mimetic S588E mutant exhibited accelerated turnover and higher polyubiquitylation levels, even in the absence of DAPK2. Conversely, the phospho-defective S588A mutant was more stable than wild-type HSPA5 (Fig. 5j and k). These findings demonstrate that DAPK2 phosphorylates HSPA5 at serine 588, which targets HSPA5 for ubiquitin-mediated proteasomal degradation.

### DAPK2-Mediated Destabilization of HSPA5 is Required for IRE1α Activation

**Fig. 6:**
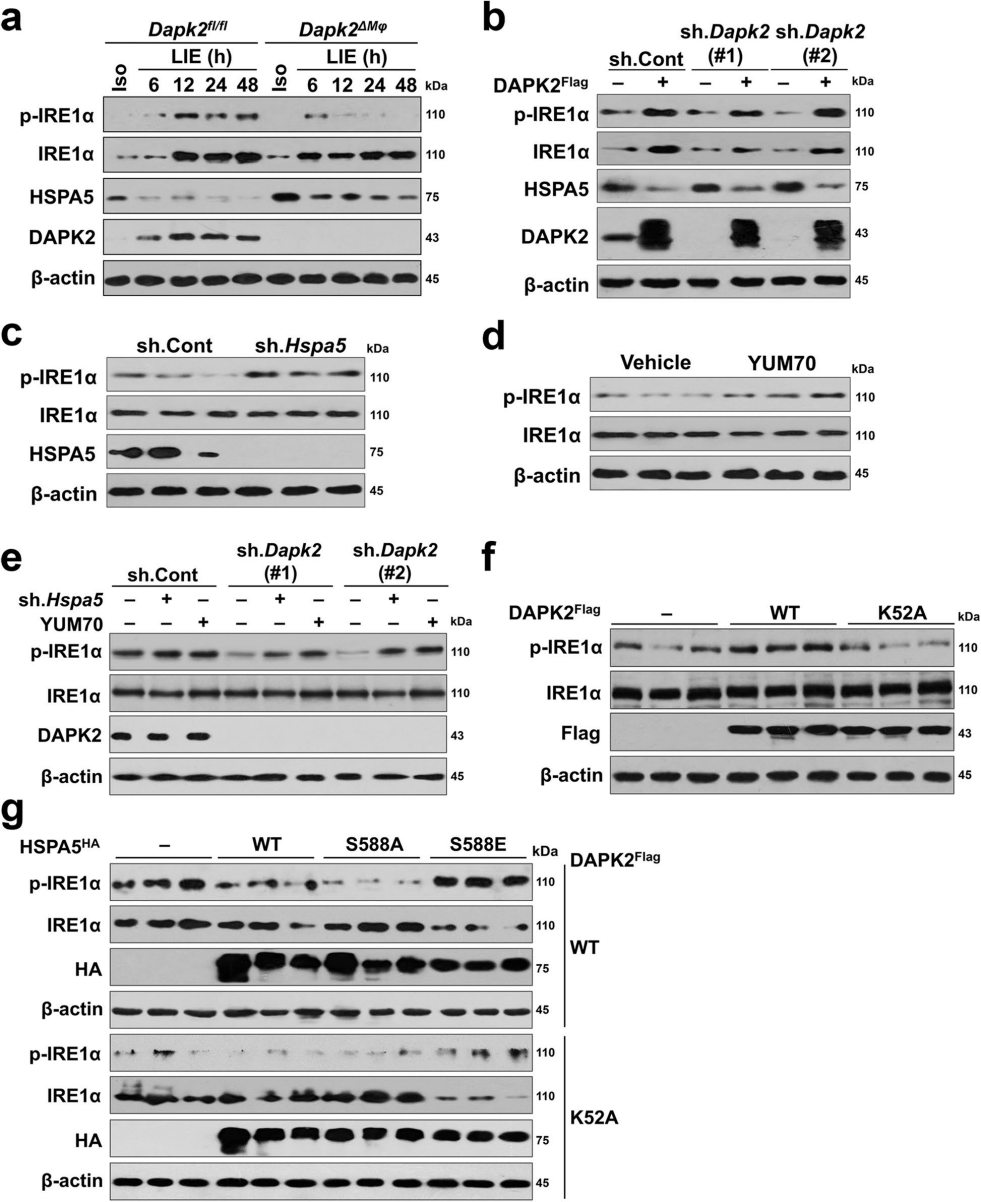
Destabilization of HSPA5 by DAPK2 leads to IRE1α activation. **a** Immunoblotting of IRE1α phosphorylation in PMs of *Dapk2*^*ΔMφ*^ versus *Dapk2*^*fl/fl*^ mice following LIE challenge at the indicated times. h, hour; Iso, isotype control; LIE, LPS-induced endotoxemia. **b** Immunoblotting of IRE1α phosphorylation in HEK293T cells transfected with short hairpin RNA (shRNA) targeting control (sh.Cont) or DAPK2 (sh.*Dapk2*) in the presence or absence of Flag-tagged wild-type DAPK2 (DAPK2^Flag^) expression. **c** Immunoblotting and quantification of IRE1α phosphorylation in HEK293T cells transfected with short hairpin RNA (shRNA) targeting control (sh.Cont) or HSPA5 (sh.*Hspa5*). **d** Immunoblotting and quantification of IRE1α phosphorylation in HEK293T cells treated with 2.5 mmol/L YUM70. **e** Immunoblotting of IRE1α phosphorylation in HEK293T cells transfected with short hairpin RNA (shRNA) targeting control (sh.Cont) or DAPK2 (sh.*Dapk2*) in the presence or absence of sh.*Hspa5* transfection and YUM70 treatment, respectively. **f** Immunoblotting and quantification of IRE1α phosphorylation in HEK293T cells transfected with Flag-tagged wild-type DAPK2 (WT) or Flag-tagged K52A mutant DAPK2 (K52A) **g** Immunoblotting and quantification of IRE1α phosphorylation in HEK293T cells transfected with Flag-tagged wild-type DAPK2 (WT) or Flag-tagged K52A mutant DAPK2 (K52A) in the presence or absence of HA-tagged wild-type HSPA5 (WT), mutant HSPA5 Ser588E (S588E) and Ser588A (S588A) reconstitution

HSPA5 is known to sequester and inactivate the ERS sensor IRE1α [21]. We therefore hypothesized that DAPK2 activates IRE1α by destabilizing HSPA5. In PMs from LIE-challenged *Dapk2*^*ΔMφ*^ mice which had higher HSPA5 levels, IRE1α phosphorylation—a marker of its activation—was robustly decreased compared with that in macrophages from *Dapk2*^*fl/fl*^ mice (Fig. 6a). Depleting DAPK2 blocked IRE1α phosphorylation, an effect that was rescued by re-expression of DAPK2 (Fig. 6b). Conversely, genetic silencing or pharmacological inhibition of HSPA5 increased basal IRE1α phosphorylation (Fig. 6c and d) and abrogated the reduction in IRE1α phosphorylation caused by DAPK2 depletion (Fig. 6e). Forced expression of wild-type DAPK2, but not the K52A kinase-dead mutant, activated IRE1α (Fig. 6f). Reconstitution with either wild-type HSPA5 or the stable S588A mutant abrogated DAPK2-mediated activation of IRE1α. In contrast, the unstable phospho-mimetic S588E mutant failed to block this activation (Fig. 6g). Therefore, DAPK2-mediated destabilization of HSPA5 is required for the activation of IRE1α.

### The HSPA5–IRE1α Axis mediates the detrimental role of macrophage DAPK2 in Sepsis

**Fig. 7:**
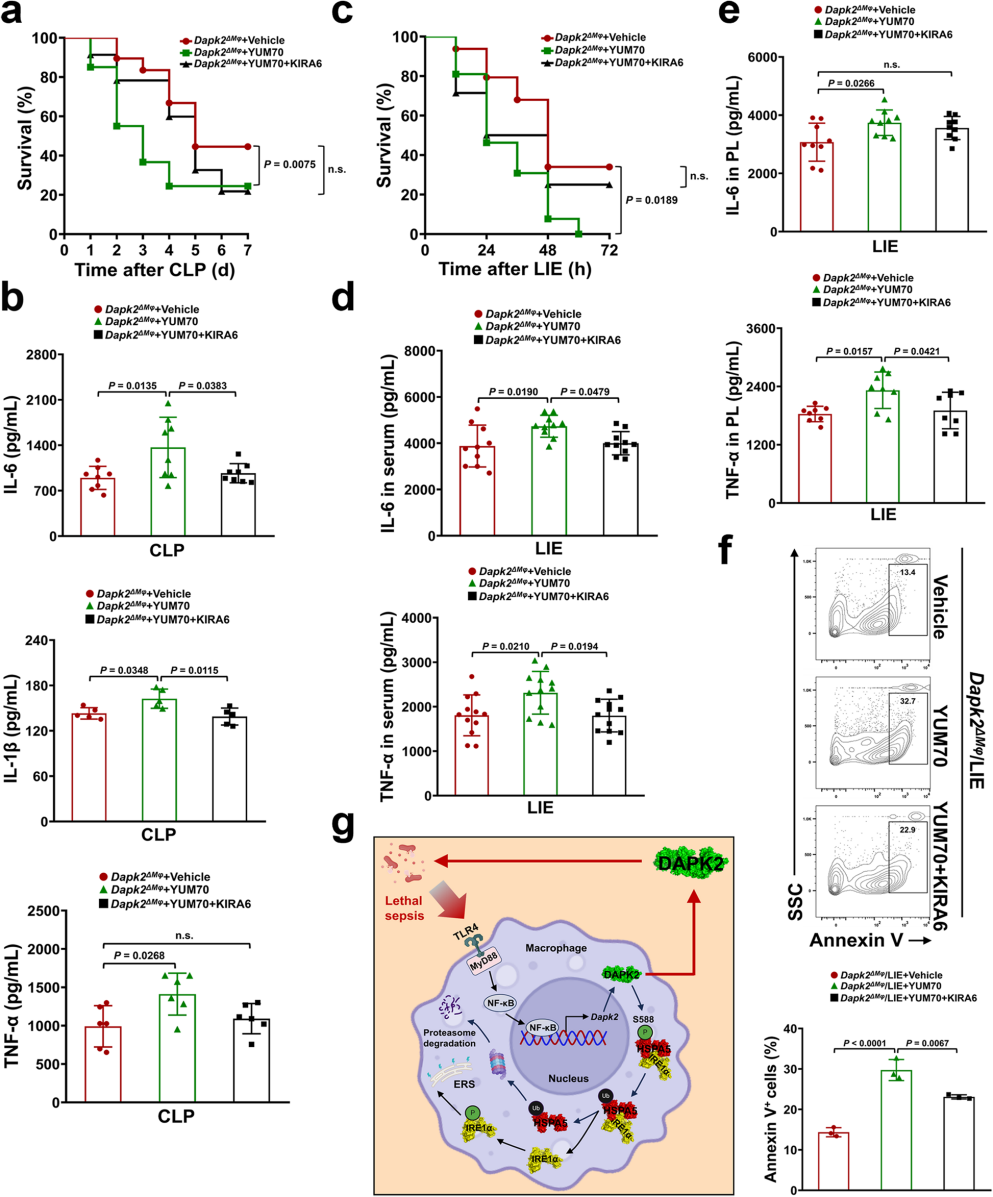
HSPA5-IRE1α axis contributes to the detrimental role of macrophage DAPK2 in sepsis. **a** Kaplan–Meier curves comparing survivals of *Dapk2*.^*ΔMφ*^ mice receiving vehicle, YUM70 treatment and YUM70 plus KIRA6 cotreatment at the indicated times after cecal ligation and puncture (CLP) operation (*n* ≥ 12 per group). d, day; n.s., no significant. **b** Enzyme-linked immunosorbent assay (ELISA) assays measuring serum levels of interleukin-6 (IL-6), interleukin-1β (IL-1β) and tumor necrosis factor-α (TNF-α) in cecal ligation and puncture (CLP)-operated *Dapk2*^*ΔMφ*^ mice receiving vehicle, YUM70 treatment and YUM70 plus KIRA6 cotreatment (*n* ≥ 5 per group). **c** Kaplan-Meier curves comparing survivals of *Dapk2*^*ΔMφ*^ mice receiving vehicle, YUM70 treatment and YUM70 plus KIRA6 cotreatment at the indicated times after LPS-induced endotoxemia (LIE) challenge (*n* ≥ 12 per group). h, hour; n.s., no significant. **d** Serum levels of interleukin-6 (IL-6) and tumor necrosis factor-α (TNF-α) in LPS-induced endotoxemia (LIE)-challenged *Dapk2*^*ΔMφ*^ mice receiving vehicle, YUM70 treatment and YUM70 plus KIRA6 cotreatment (*n* ≥ 10 per group). **e** Enzyme-linked immunosorbent assay (ELISA) assays detecting levels of interleukin-6 (IL-6) and tumor necrosis factor-α (TNF-α) in peritoneal lavage fluid of LPS-induced endotoxemia (LIE)-challenged *Dapk2*^*ΔMφ*^ mice receiving vehicle, YUM70 treatment and YUM70 plus KIRA6 cotreatment (*n* ≥ 8 per group). n.s., no significant.** f** Contour plots and quantification of flow cytometry with Annexin V staining in PMs of LPS-induced endotoxemia (LIE)-challenged *Dapk2*^*ΔMφ*^ mice receiving vehicle, YUM70 treatment and YUM70 plus KIRA6 cotreatment (*n* = 3 per group). **g** Schematic diagram summarizing the molecular mechanism through which DAPK2 propagates ERS of macrophages in sepsis**.** Data are expressed as mean ± s.d. (b and d-f). Log-rank t test (a and c) and Two-sided ANOVA with Bonferroni post hoc *t* test correction (b-f) and was used to calculate the *P* value, respectively

To determine the causal link between the DAPK2–HSPA5–IRE1α axis and sepsis pathogenesis in vivo, we used a pharmacological strategy in *Dapk2*^*ΔMφ*^ mice. Administration of YUM70, a selective inhibitor of HSPA5, significantly shortened the survival of CLP-operated *Dapk2*^*ΔMφ*^ mice, effectively reversing their survival advantage. This detrimental effect of HSPA5 inhibition was completely abrogated by co-administration of KIRA6, a selective IRE1α kinase inhibitor (Fig. 7a). Similarly, YUM70 exacerbated the cytokine storm in CLP-operated *Dapk2*^ΔMφ^ mice, an effect that was nullified by KIRA6 (Fig. 7b). YUM70 also increased macrophage death, which was reversed by KIRA6 (Fig. S5a). Consistent results were observed in the LIE model. YUM70 treatment of *Dapk2*^*ΔMφ*^ mice increased mortality, inflammatory cytokine production, and lung injury after LIE challenge; these effects were all reversed by co-treatment with KIRA6 (Fig. 7c-e and S5b). Furthermore, the increased macrophage death induced by YUM70 in LIE-challenged *Dapk2*^*ΔMφ*^ mice was prevented by co-administration of KIRA6 (Fig. 7f). Collectively, these results demonstrate that the detrimental effects of macrophage DAPK2 in sepsis are mediated through the HSPA5–IRE1α signaling axis.

## Discussion

Endoplasmic reticulum stress (ERS) is associated with excessive inflammation, programmed cell death, and organ damage elicited by both microbial infection and sterile insults [35, 36]. In this study, we demonstrate that death-associated protein kinase 2 (DAPK2) triggers the phosphorylation of HSPA5 at serine 588 and its subsequent proteasomal degradation, leading to the activation of IRE1α. This process propagates macrophage ERS and exacerbates sepsis (Fig. 7g). Our findings provide new insights into the mechanisms underlying macrophage ERS during systemic infection and identify a novel therapeutic target for sepsis. Given the incomplete understanding of the pathophysiology of sepsis and the complexity of the factors involved, future work should aim to ascertain whether specific components of the inflammatory-immune network correspond to particular pathogenic virulence and to determine whether pharmacological targeting of DAPK2 can protect against sepsis through modulation of the HSPA5–IRE1α axis.

Alterations in metabolic responses following systemic infections have been considered to be essential for an appropriate host immunity and macrophage fate. Early study demonstrated that inflammatory macrophages accumulate more succinate and tend to be more glycolytic than the resting macrophages [37]. Pharmacological inhibition of aerobic glycolysis can prohibit release of inflammatory cytokines in macrophages and improve survival of sepsis in mice [38]. While able to initiate inflammatory response through TLRs, pathogen lipids accelerate development and progression of septic shock [39]. Prospective study with plasma metabolomics and proteomics revealed that fatty acid oxidation (FAO) is restricted in sepsis non-survivors [40]. For this reason, the lack of FAO is presumably associated with poor clinical outcome of septic patients [41]. ERS favors mitochondrial bioenergetics, effectively achieving this through catalyzing respiratory supercomplexes (SCs) formation and adenosine triphosphate (ATP) synthesis [42]. Moreover, previous studies have shown that ERS suppresses FAO as an important source of lipid accumulation in parenchymal tissues [43]. These evidence and hints probably explain why DAPK2 becomes critical for inflammatory phenotypes and macrophage death during sepsis.

Our study provides evidence supporting the concept that DAPK2 loss-of-function can prevent cell apoptosis [44, 45]. Our data demonstrate that DAPK2 is highly expressed in macrophages from septic patients and mice, and its expression can be transcriptionally upregulated by pro-inflammatory TLR4/MyD88/NF-κB signaling. In our model, macrophage-specific DAPK2 deficiency reduces septic lethality, the associated cytokine storm, and macrophage death. We did not observe any effect of mTORC1 on the detrimental role of DAPK2, nor was DAPK2 implicated in the phenotypic plasticity of macrophages. Although the evidence presented here indicates that DAPK2 exacerbates sepsis, it is undoubtedly one of numerous factors governing this complex process. Therefore, it is likely that integrative molecular and genetic pathways coordinate DAPK2-mediated macrophage death and excessive inflammation.

DAPK2 exerts its biological functions through phoshorylating substrate proteins [15]. To identify the downstream effector of DAPK2 responsible for macrophage ERS, we identified HSPA5 using co-immunoprecipitation (co-IP) followed by liquid chromatography with tandem mass spectrometry (LC–MS/MS). Subsequent predictive analysis with AlphaFold-Multimer revealed several residues that may be required for the intermolecular interaction between the two proteins. We demonstrate that the serine 588 residue, located outside of the HSPA5 nucleotide-binding domain (NBD) and substrate-binding domain (SBD), is a conserved phosphorylation site for DAPK2. Although the precise functions of the NBD and SBD are not fully understood, these domains have been linked to adenosine triphosphate (ATP) hydrolysis and the unfolded protein response (UPR) [46]. While it remains undefined whether phosphorylation at serine 588 influences the activity of the NBD or SBD, this modification appears to accelerate the proteasomal degradation of HSPA5. To our knowledge, this is the first study to report the role of DAPK2 in triggering HSPA5 phosphorylation and proteasomal degradation in macrophages. It has been previously noted that destabilization of HSPA5 is critical for cytotoxic ERS and apoptosis [47]. These findings suggest an opportunity to explore alternative molecules upstream of HSPA5 to better understand the biology of ERS in systemic infection.

Our finding that HSPA5 is required for DAPK2-inducible IRE1α activation is significant. IRE1α, together with two other transmembrane receptors—activating transcription factor 6 (ATF6) and protein kinase RNA-like endoplasmic reticulum kinase (PERK)—is traditionally recognized as a cytosolic sensor of the UPR [48]. Under physiologic conditions, IRE1α is sequestered by HSPA5 and maintained in an inactive, monomeric state. The dissociation of HSPA5 from IRE1α leads to autophosphorylation and activation of IRE1α, which in turn contributes to ERS. Evidence suggesting that IRE1α might fulfill pro-inflammatory functions in addition to sensing the UPR derives from studies showing that IRE1α activation compromises innate immune host defense during infection with *Staphylococcus aureus* (MRSA), a pathogen that triggers ERS in pneumonia [49]. Furthermore, IRE1α can mediate ERS-triggered inflammation in macrophages through nucleotide-binding and oligomerization domain (NOD)-, leucine-rich repeat (LRR)- and pyrin domain-containing protein 3 (NLRP3)–dependent mitochondrial damage [50]. Our results are consistent with these findings and emphasize that IRE1α activation, resulting from the DAPK2-mediated proteasomal degradation of HSPA5, exacerbates the pathogenic processes of sepsis.

This study has several limitations. Our mechanistic studies regarding HSPA5 polyubiquitylation in human or murine cells underline the fact that the DAPK2-mediated degradation of HSPA5 has been delineated. Nonetheless, we do not identify the downstream effector of DAPK2 that serves as the bona fide E3 ubiquitin ligase of HSPA5. Several E3 ligases, including autocrine motility factor receptor (AMFR) [51], tripartite motif containing 7 (TRIM7) [52] and mitochondrial E3 ubiquitin protein ligase 1 (MUL1) [53] were shown to catalyze the K48-linked poly-ubiquitylation and degradation of HSPA5, whereas the role of specific E3 ligase such as tripartite motif containing 38 (TRIM38) seems to be different [54], with poor understanding of the relationship between HSPA5 ubiquitylation and biological function. It is tempting to speculate that HSPA5 degradation by DAPK2 might involve distinct regulatory E3 ligases. Although our data clearly demonstrate that DAPK2 phosphorylates HSPA5 at serine 588, the possibility that DAPK2 phosphorylates other serine residues of HSPA5 cannot be ruled out. Moreover, the specific domains responsible for the physical interaction between DAPK2 and HSPA5 have not been delineated. A definitive conclusion regarding this intermolecular interaction will require crystal structure analysis. Furthermore, we did not address whether pharmacological targeting of DAPK2 modulates ERS of macrophages in sepsis. Currently, no specific DAPK2 inhibitor is available for either clinical or experimental use. A compound with a pyrazolo [1,5-*a*] pyrimidine-3- carbonitrile scaffold that target casein kinase (CK2) has been developed as the co-inhibitor of DAPK-2 and −3 kinases [55]. A major future task will be to demonstrate the therapeutic efficacy of targeting DAPK2 in murine models of sepsis.

Our vital finding has implications for proof-of-concept standpoint that targeting DAPK2 as a viable anti-septic therapeutic approach. However, since our work was done in macrophage-deficient mice models, whether inflammation-relevant ERS propagation occurs in other cell types is needed to be scrutinized. Albeit a constructed framework in our study, we, like most other scholars, made conclusions under background of genetic DAPK2 loss. As non-immune cells that are inflammation regulatory and DAPK2 expression likely exist ubiqutiously, it is conceivable that DAPK2 may also boost ERS in multiple cell types. In addition, when variant is present, the kinetics of DAPK2 signaling cascade likely differ and may lead to disparate conclusions. Further studies should be performed using knockin DAPK2 polymorphic variants mice, and the benefits of cell-specific shRNA delivery or transient inhibition remain to be determined.

In conclusion, we demonstrate that DAPK2 triggers the phosphorylation and proteasomal degradation of HSPA5 to activate IRE1α, thereby propagating ERS in macrophages and exacerbating sepsis. Our study identifies a promising therapeutic target and lays the framework for developing novel therapeutic strategies for the prevention of this ongoing public health crisis.

## Methods

### Reagents and antibodies

The following reagents were used in this study: *Escherichia coli* O111:B4 LPS-EB (tlrl-3pelps, InvivoGen), YUM70 (HY-138364, Medchem Express), Pam3CSK4 (HY-P1180, Medchem Express), rapamycin (R-5000, LC laboratories), BMS345541 (HY-10519, Medchem Express), JSH-23 (HY-13982, Medchem Express), cycloheximide (SBR00013, Sigma-Aldrich), MG132 (M7449, Sigma-Aldrich), chloroquine (C6628, Sigma-Aldrich), KIRA6 (S8658, Selleck Chemicals LLC), FAM-FLICA (9145, ImmunoChemistry), RNeasy kit (75,162, Qiagen), RPMI 1640 (11,875,119, Gibco), penicillin–streptomycin (15,070–063, Gibco) and nickel charged magnetic agarose beads (36,113, Qiagen). The following antibodies were used in immunoprecipitation and immunoblotting: anti-HA (26,183, Invitrogen), anti-Flag (F7425, Millipore), anti-V5 (R960-25, Invitrogen), anti-pSer (sc-81514, Santa Cruz), anti-DAPK2 (NBP2-02477, Novus Biologicals), anti-pIRE1α (NB100-2323, Novus Biologicals), anti-pMLKL (37,333, Cell Signaling Technology), anti-cleaved caspase-3 (9661, Cell Signaling Technology), anti-GSDMD p30 (AF3127, Beyotime), anti-β-actin (bsm-33036 M, Biosynthesis), anti-HSPA5 (66,574–1-Ig, Proteintech Group, Inc.), anti-Ub (10,201–2-AP, Proteintech Group, Inc.), anti-GST (sc-138, Santa Cruz) and anti-IRE1α (ab37073, Abcam).

### Lentiviral transfection

Lentiviral transfection of shRNAs, sg.RNA and plasmids were performed as previously described [56–59]. In brief, HEK293T cells were cotransfected with 350 ng of lentiviral pLKO.1-Puro shRNA plasmids and packaging shRNA plasmids (pHDM-VSV-G, pHDM-tat1b, pHDM-HgPM2 and pRC-CMVRaII). Next day medium was replaced by Dulbecco's modified Eagle's medium (DMEM) containing 20% heat-inactivated fetal bovine serum (FBS) and supernatants were collected 72 h later, pooled and passed through a 0.45-μm filter. After packaging, lentivirus infection was conducted by 1 mL viral supernatant with the addition of 8 μg/mL polybrene (TR-1003, Sigma-Aldrich) for 48 h. Transfectants were selected and expanded with puromycin (P-600–500, Gold Biotechnology) at 4 μg/mL and monoclones were picked up for subsequent analyses. For the CRISPR-Cas9-mediated knockout of TLR4, a total of 1 × 10^5^ THP-1 macrophages were transduced with LentiGuide-Puro-sg.*Tlr4* lentiviral supernatants produced by cotransfected HEK293T cells with 2 μg gRNA plasmids and packaging plasmids (800 ng pVSVg and psPAX2). Cells were selected with 1 μg/mL puromycin for 2 days.

### Cell culture and stimulation

Human THP-1 monocytes (TIB-202) were obtained from American Type Culture Collection (ATCC) and were grown in RPMI-1640 medium supplemented with 10% fetal bovine serum (FBS) at 37 °C with 5% CO2 and 95% air. All cells were allowed to differentiate into macrophages for 24 h using 100 nmol/L PMA (P1585, Sigma-Aldrich). Primary mouse peritoneal macrophages (PMs) were harvested from the peritoneal cavity of C57BL/6 J mice by puncture with injection of 5 mL of cold PBS, 2 mmol/L ethylenediaminetetraacetic acid (EDTA) and cultured in Roswell Park Memorial Institute (RPMI) 1640 medium supplemented with 10% FBS and 2 mmol/L glutamine. Femurs from C57BL/6 J mice at 6–10 weeks of age were utilized to generate bone-marrow-derived macrophages (BMDMs), which were cultured in DMEM (Gibco) containing 20% L929-conditioned media, 10% FBS and 100 U/mL penicillin–streptomycin, and allowed to differentiate in the presence of 20 ng/mL recombinant mouse granulocyte macrophage colony-stimulating factor (GMCSF) for 7 days. THP-1 macrophages were stimulated with agents at the following concentrations unless otherwise indicated: 1 µg/mL of LPS, 30 µmol/L BMS345541 and 30 µmol/L JSH-23. For BMDMs, 400 ng/mL of LPS and 1 µg/mL of Pam3CSK4 were used. To block protein synthesis, HEK293T cells were treated with 100 µg/mL of cycloheximide for the indicated times. Proteasome and lysosome was inhibited by 20 µmol/L of MG132 and 100 μmol/L of chloroquine, respectively.

### Animal studies

Murine models of the sterile and polymicrobial sepsis were established as described previously [56, 60, 61]. In general, LPS-induced endotoxemia (LIE) was established by intraperitoneal injection of 20 mg/kg LPS from *Escherichia coli* O111:B4 (InvivoGen) into mice, which were then put back in a temperature-controlled environment freely accessible to water and food and were monitored for survival. At 48 h after sepsis induction except where indicated otherwise, mice were euthanized, then lung tissues were randomly and blindingly harvested for evaluating histopathology. For cecal ligature and puncture (CLP), mice were anesthetized with ketamine (100 mg/kg) and a midline abdominal incision was made, after that, the cecum was punctured using a 17-gauge needle. Mice in sham group experienced anesthesia, laparotomy and abdomen closure without CLP. *Dapk2 *^*fl/fl*^ (S-CKO-01992) mice were obtained from Cyagen Biosciences. *Dapk2*^*ΔMφ*^ mice were generated by crossing *Dapk2 *^*fl/fl*^ mice with mice harboring Cre recombinase under control of *Lyz2* promoter. Littermates without Cre but with floxed alleles were served as controls. All mice were bred in specific pathogen-free (SPF) conditions under a 12 h/12 h light/dark cycle and were fed standard chow. Mice in each experimental group were sex- and age-matched for in vivo studies. To calculate lung wet to dry ratio, fresh lungs were excised after removing the blood and weighed to measure the wet weight, followed by obtaining the constant dry weight in an 80 °C oven. To examine the effect of blockade of mTORC1 pathway, *Dapk2*^*ΔMφ*^ mice were subjected to LIE challenge and randomly divided into two groups: the vehicle groups were received intraperitoneally (i.p.) injection of PBS at 3 and 15 h post-LIE, respectively, and the rapamycin groups were received i.p. injection of 150 µg rapamycin at the same time points post-LIE. To evaluate the efficacy of targeting HSPA5, YUM70 (30 mg/kg body weight) was i.p. injected into mice 6 h after onset of CLP or LIE. To deactivate IRE1α, 10 mg/kg of KIRA6 was i.p. injected into mice in combination with YUM70. For evaluation of hepatic function, approximately 0.5 mL blood was collected from venae angularis of the indicated mice. After incubation on ice for half an hour, blood samples were centrifuged at 3000 g for 10 min to collect serum. serum ALT was measured by commercially available colorimetry kit (A11A01627 for ALT) from HORIBA ABX SAS according to the manufacturer’s guidelines. All animal experiments were approved by the Institutional Animal Care and Use Committee of Zhejiang Provincial People's Hospital and performed in accordance with the National Institutes of Health (USA) guidelines.

### Protein identification by liquid chromatography-tandem mass spectrometry (LC–MS/MS)

PMs was isolated from the LIE-challenged *Dapk2*^*ΔMφ*^ mice, lysed with radioimmunoprecipitation assay (RIPA) buffer (P0013B, Beyotime) containing protease and phosphatase inhibitors and then incubated with the His-tagged DAPK2 protein, followed by a pulldown assay using nickel-nitrilotriacetic acid (NTA) magnetic agarose beads (Qiagen). After that, the eluted samples were separated in sodium dodecyl sulfate (SDS)-polyacrylimide gel (PAGE) and visualized by Coomassie brilliant blue staining. The protein bands were cut and washed thrice with NH_4_HCO_3_ (A6141, Sigma-Aldrich) and acetontrile (360,457, Sigma-Aldrich), and digested with sequencing-grade modified trypsin (V5111, Promega) containing RapiGest SF (186,002,123, Waters Corporation) in ammonium bicarbonate buffer at 37 °C overnight. LC–MS/MS was performed on a Q-Exactive mass spectrometer (Thermo Fisher Scientific) for 90 min. MS spectra were searched using Proteome Discoverer (v2.1) based on *Mus musculus* proteome database (UniProt) and Scaffold Local FDR algorithm was used to validate the identified proteins.

### GST-pulldown assay

GST-pulldown assay was performed as described previously [16]. The full-length human DAPK2 was subcloned into pGEX-4 T-1 vector (129,572, Addgene) with N-terminal GST tag. Vectors were transformed into BL21 (DE3) *E. coli* (EC0114, Thermo Fisher Scientific) and single-colony was cultured with 100 μg/mL ampicillin and 0.5 μM isopropylthiogalactoside for 18 h. The bacterial pellet was lysed in NETN buffer (0.5% NP40, 20 mM Tris at pH 8.0, 100 mM NaCl and 1 mM EDTA), followed by centrifugation at 20,000 r.p.m. for 1 h at 4 °C and filtration with a 0.45-μm syringe filter. Then Glutathione Sepharose beads (G4510, Millipore) were incubated with lysates at 4 °C overnight. The purified GST-DAPK2 protein was washed three times with lysis buffer and incubated with HA-HSPA5 for 2 h on ice. Reactions were terminated by boiling at 95 °C with SDS sample buffer for 10 min and analyzed by immunoblotting.

### Co-immunoprecipitation (co-IP) and immunoblotting

co-IP and immunoblotting were performed as described previously [16, 62]. For co-IP assay, cells were lysed in RIPA buffer with 0.1% SDS, 1 mM phenylmethylsulfonyl fluoride, 1 mM sodium orthovanadate (Na_3_VO_4_) and 10 mM sodium fluoride (NaF). After rotation for 30 min at 4 °C, After centrifugation at 15,000 r.p.m. for 10 min, supernatants of the lysates were transferred to the fresh tubes. Immunoprecipitation was performed using the indictaed primary antibodies with rotation with protein A-agarose beads (16–125, Sigma-Aldrich) overnight at 4 °C. The immunoprecipitated proteins were rinsed with wash buffer and resolved by SDS-PAGE. For immunoblotting, cells were lysed in 50 mM Tris–HCl (pH 7.4), 150 mM NaCl, 1 mM EDTA and 1% NP-40 lysis buffer containing protease and phosphatase inhibitors with five sonication cycles per sample. After incubation on ice for 20 min, lysates were centrifuged at 15,000 r.p.m. for 10 min to collect supernatants, after which protein concentration was quantified using Pierce™ BCA Protein Assay Kit (23,225, Thermo Fisher Scientific). The quantified protein (40 µg) was boiled in 5 × Laemmli buffer with 315 mM Tris–HCl (pH 6.8), 0.0001% Bromphenol blue, 10% SDS, 50% Glycerol and 500 mM DTT, fractionated on 10% SDS-PAGE and transferred to polyvinylidene difluoride membranes (Immobilon™). The membrane was blocked in 5% bovine serum albumin (BSA) for 1 h and incubated with the primary antibodies diluted in TBST (0.1% Tween20 and Tris-Buffered Saline) at 4 °C overnight. Next day the horseradish peroxidase (HRP)-conjugated secondary antibodies were probed for 1 h at room temperature. Protein bands were visualized by western chemiluminescent HRP substrate kit (P1000-100, PPLYGEN). Analysis and quantifications of protein expression were performed using ImageJ software (National Institutes of Health). Data are presented as mean and error bars as standard deviation of at least three replicates.

### Cellular ubiquitination assay

Cellular ubiquitination assay were carried out as described previously [16, 62]. For HSPA5 ubiquitination, 4 × 10^5^ of HEK293T cells or 2 × 10^7^ of PMs were lysed in RIPA buffer containing 50 mM Tris HCl (pH 7.4), 150 mM NaCl, 1 mM EDTA, 1% Nonidet P-40, protease, phosphatase inhibitors and 10% glycerol. Lysates were boiled in 1% SDS at 95 °C for 10 min, followed by centrifugation at 15,000 r.p.m. for 5 min and incubation with an anti-HSPA5 antibody (Proteintech Group, Inc.) at 4 °C overnight. Next day protein A-agarose beads (16–125, Millipore) were incubated with lysates for 2 h at 4 °C, washed and resuspended with Pierce Elution Buffer (21,009, Thermo Fisher Scientific) containing 4 × SDS sample buffer. Levels of HSPA5 ubiquitination were determined by immunoblotting analyses using antibody to Ub in the immunoprecipitated lysates. In nickel-NTA-agarose his-ubiquitin purification experiments, 5 × 10^6^ of HEK293T cells were transfected with His-Ub vectors and various constructs as indicated. At 48 h post-transfection, cells were lysed in denatured buffer (6 M guanidine HCl, 0.1 M Na_2_HPO_4_/NaH_2_PO_4_, 0.01 M Tris–HCl [pH 8.0] and 10 mM bmercaptoethanol). The lysate was sonicated for 15 s and mixed with 50 µL of nickel-nitrilotriacetic acid (NTA) magnetic agarose beads (36,113, Qiagen) on a rotator at room temperature for 3 h. The beads were washed three times with denatured buffer and diluted in 25 mM Tris–HCl (pH 6.8) and 20 mM imidazole. The eluted proteins were resolved in SDS-PAGE and analyzed by immunoblotting using anti-HA antibody.

### In vitro kinase assay

In vitro kinase assay was performed as reported previously [16]. In brief, 2.5 μg of V5-tagged HSPA5 protein was incubated with 100 ng of active DAPK2 (SRP5018, Sigma-Aldrich) in a kinase buffer containing 20 µM adenosine triphosphate (ATP) at 30 °C for 30 min. Reaction products were separated by SDS-PAGE and phosphorylation was examined using an anti-pSer antibody.

### Cycloheximide (CHX) pulse-chase experiments

CHX pulse-chase experiments were carried out as described previously [63]. The detailed information was provided in Supplemental Materials and Methods.

### Luciferase reporter assays

The procedures of luciferase assays were described in previous publications [64, 65]. The detailed information was provided in Supplemental Materials and Methods.

### RNA extraction and real-time quantitative PCR (RT-qPCR)

RT-qPCR was performed as described previously [56, 60]. Total RNA was prepared using an RNeasy Kit (Qiagen), followed by cDNA synthesis using a SuperScript™ VILO™ cDNA synthesis kit (11,754,250, Invitrogen). Real-time quantitative PCR was performed on an Applied Biosystems 7900HT cycler using Power SYBR Green PCR Master Mix (RR420A, Takara). Thermal cycling conditions were: 95 °C for 10 s, 40 cycles of denaturing 95 °C for 5 s, annealing 55 °C for 10 s and extension 72 °C for 15 s. The primers used for RT-qPCR were listed as below: human *Dapk2* sense, 5′-CATCCTTGAGCTAGTGTCTGGA-3′ and *Dapk2* antisense, 5′-GGATCTGCTTAATGAAGCTGGT-3′; mouse *Dapk2* sense, 5′-CCTCGATGAGG.

AGCCCAAATA-3′ and *Dapk2* antisense, 5′-CCCGGCACTTCTTCACGAT-3′;

human *Hspa5* sense, 5′-CATCACGCCGTCCTATGTCG-3′ and.

*Hspa5* antisense, 5′-CGTCAAAGACCGTGTTCTCG-3′. The amounts of individual transcript were normalized to those of β-actin.

### Transmission electron microscopy (TEM)

TEM was performed as previously described [57]. In brief, PMs were isolated from mice as indicated and cultured in 24-well plates prior to fixation with 0.5 mol/L sodium cacodylate buffer containing 2% glutaraldehyde at 4 °C for 1 h, followed by secondary fixation with 1% osmium tetroxide plus 1% potassium ferrocyanide at 4 °C for 1 h. After being dehydrated by ethanol, cells were infiltrated in a 1:1 mixture of absolute ethanol and EMbed 812 epoxy resin, and then embedded. A Reichert-Yung Ultracut ultramicrotome was used to collect ultrathin sections, which were counterstained with lead citrate and uranyl acetate subsequently. Images were taken on a Hitachi H7700 electron microscope.

### Enzyme-linked immunosorbent assay (ELISA)

ELISA kits were used to measure the levels of mouse IL-6 (555,240, BD Biosciences), IL-1β (432,604, Biolegend), HMGB1 (ARG81310, Arigo) and TNF-α (555,268, BD Biosciences) in serum and peritoneal lavage fluid according to the manufacturer’s recommendation. In brief, MaxiSorp 96-well plates (44–2404, Thermo Fisher Scientific) was coated with capture antibody overnight at 4 °C in 15 mM Na_2_CO_3_ and 35 mM NaHCO_3_ (pH 9.6), washed thrice by PBS and blocked with 5% BSA at 37 °C. Thirty minutes later, 100 µL of sera and standard samples were added into wells and incubated at room temperature for 2 h, followed by three washes with PBST (0.1% Tween-20 in PBS). Afterwards, the biotin-conjugated antibody was added into wells and incubated at 37 °C for 2 h. HRP-streptavidin was incubated for 30 min at room temperature after three washes with washing buffer. Signals of ELISA were detected at 450 nm.

### Immunofluorescene (IF) staining

Immunofluorescene (IF) staining was conducted as described previously [59, 60, 62]. The detailed information was provided in Supplemental Materials and Methods.

### Fluorescence activating cell sorter (FACS) analysis

FACS for surface and intracellular staining was performed as previously described [56, 57]. In brief, BMDMs were incubated with 100 ng/mL of LPS plus 100 U/mL of IFN-γ or 10 ng/mL of IL-4 for 12 h and stained with the primary antibodies against surface markers F4/80 (123,110, BioLegend), CD11c (117,310, BioLegend) and CD206 (141,708, BioLegend). Cell death was determined by flow cytometry with Annexin V staining. Briefly, PMs isolated from peritoneal fluid of mice were resuspended in 1 mL ACK lysis buffer (R7757, Sigma-Aldrich) for 3 min. After centrifugation and a washing step, PMs were suspended in 50 µL PBS containing 1% FBS and stained with 25 μg/mL Annexin V-FITC (C1062M, Beyotime) at room temperature for 1 h. FACS analysis was performed with a BD LSR II flow cytometer (BD Biosciences). For intracellular staining, cells were permeabilized with 0.2% saponin at 25 °C for 30 min, washed twice with PBS and then incubated with the monoclonal anti-DAPK2 antibody (NBP2-02477, Novus Biologicals) on ice for 20 min. The cells were washed with buffer and subjected to FACS. The flow data were analyzed and acquired by FlowJo VX software (Tree Star Inc.).

### Histopathology scoring

Lungs were excised, fixed with 10% formalin and paraffin-embedded by standard procedures. Hematoxylin and eosin (H&E) staining was performed on 5-µm sections with examination by a pathologist blinded to experiments. For morphology evaluation, at least seven random visual fields were chosen under microscopy. The severity of inflammation was counted on a semiquantitative scale of 0 to 4: 0, normal morphology; 1, mild (1–10%); 2, moderate; 3, severe; 4, almost all (≥ 75%).

### Statistical analysis

Statistical analysis was performed with GraphPad Prism software (La Jolla, CA, USA). Kaplan–Meier curves and log-rank test were used to analyze survival data. Difference between two groups was conducted with unpaired two-tailed Student’s *t* tests, and one-way analysis of variance (ANOVA) followed by Bonferroni post hoc *t* test correction was used for multiple group comparisons. Data were expressed as means ± standard deviation (s.d.). The *P* value of less than 0.05 was considered statistically significant.

## Supplementary Information

Supplementary Material 1.

## Data Availability

Human RNA sequencing (RNA-seq) and single-cell RNA sequencing (scRNA-seq) datasets from control and sepsis groups were sourced from the Gene Expression Omnibus (GEO), including GSE57065 and GSE167363. The authors declare that all data supporting the findings in this study are available within the paper. All data are available from the authors upon reasonable request.

## Abbreviations

ERS: Endoplasmic reticulum stress
IRE1α: Inositol-requiring enzyme 1 α
DAPK2: Death-associated protein kinase 2
TLR4: Toll-like receptor 4
MyD88: Myeloid differentiation primary response gene 88
HSPA5: Heat shock protein family A (Hsp70) member 5
NF-κB: Nuclear factor-κB
IL-1β: Interleukin-1β
TNF-α: Tumor necrosis factor-α
LPS: Lipopolysaccharides
SIRS: Systemic inflammatory response syndrome
PAMPs: Pathogen-associated molecular patterns
PRRs: Pattern recognition receptors
LIE: LPS-induced endotoxemia
CLP: Cecal ligature and puncture
PMs: Peritoneal macrophages
BMDMs: Bone-marrow-derived macrophages
HMGB1: High mobility group box 1
UPR: Unfolded protein response
DMEM: Dulbecco's modified Eagle's medium
FBS: Fetal bovine serum
RIPA: Radioimmunoprecipitation assay
LC-MS: Liquid chromatography-tandem mass spectrometry
co-IP: Co-immunoprecipitation
NTA: Nickel-nitrilotriacetic acid
GST: Glutathione *S*-transferase
GTEx: Genotype-Tissue Expression
GEO: Gene Expression Omnibus
HEK293T: Human embryonic kidney-293 T
scRNA-seq: Single-cell RNA-sequencing
ALT: Alanine transaminase
ELISA: Enzyme-linked immunosorbent assay
FACS: Fluorescence activating cell sorter
RT-qPCR: Real-time quantitative PCR
