# Supplementary material for: Death-associated protein kinase 2 (DAPK2) propagates endoplasmic reticulum stress in macrophages to worsen sepsis through HSPA5-IRE1α axis
Source: Mol Biomed. 2026 Jun 23;7:94. doi: 10.1186/s43556-026-00501-9 (PMC13291292; doi:10.1186/s43556-026-00501-9)

**Death-associated protein kinase 2 (DAPK2) propagates endoplasmic reticulum stress in macrophages to worsen sepsis through HSPA5-IRE1α axis**

Yin Ni^1, 2^, Guo-Zhen Tang^3, 4^, Chen Qiu^5^, Ge Zhu^6^, Shu-Wen Jin^7^, Hai-Ping Zhu^8^, Shi-Jing Mo^2, 3, 4*^, Xiang-Ming Fang^1*^

^1^*Department of Anesthesiology and Intensive Care, The First Affiliated Hospital, School of Medicine, Zhejiang University, Hangzhou 310003,* *P.R. China.*

^2^*Emergency and Intensive Care Unit Center, Intensive Care Unit, Zhejiang
Provincial People’s Hospital (Affiliated People’s Hospital), Hangzhou Medical College, Hangzhou 310014, Zhejiang, P.R.China*

^3^*Center for Rehabilitation Medicine, Department of Intensive Rehabilitation Care
Unit, Zhejiang Provincial People’s Hospital (Affiliated People’s Hospital), Hangzhou
Medical College, Hangzhou 310014, Zhejiang, P.R.China*

^4^*Center for Rehabilitation Medicine, Rehabilitation & Sports Medicine Research
Institute of Zhejiang Province, Department of Rehabilitation Medicine, Zhejiang
Provincial People’s Hospital (Affiliated People’s Hospital), Hangzhou Medical
College, Hangzhou 310014, Zhejiang, P.R.China*

^5^*Department of Ultrasound in Medicine, Second Affiliated Hospital of Zhejiang University School of Medicine,* *Hangzhou* *310003, Zhejiang, P.R.China*

^6^*Center for Veterinary Sciences, Zhejiang University, Hangzhou 310058, Zhejiang, P.R.China*

^7^*Zhejiang Lab, Hangzhou 311121, Zhejiang, P.R.China*

^8^*Department of Intensive Care Unit, The First Affiliated Hospital, Wenzhou Medical University, Wenzhou 325000, Zhejiang, P.R.China*

^*^**Corresponding author:**

Shi-Jing Mo, MD, PhD

Email: mmdoctor8637@126.com

ORCID: <https://orcid.org/0000-0002-1303-4766>

XiangMing Fang, MD, PhD

E-mail: xiangming_fang@163.com

**Supplemental Materials and Methods**

**Plasmid construction**

His-ubiquitin (His-Ub) plasmid (159326) was obtained from Addgene (Cambridge, MA). Human open reading frame (ORF) of DAPK2 (NM_014326.3) was synthesized from GenScript and subcloned to pLenti-CMV-FLAG-His (P100020, Charles River). Human HSPA5 from pDONR223_HSPA5_WT (82132, Addgene) was cloned into pLenti-C-HA-IRES-BSD vector (PS100104, Origene) with C-terminal hemagglutinin (HA) tag or pLX304 vector (25890, Addgene) with C-terminal V5 tag using MultiSite Gateway^TM^ Pro Plus (12537100, Thermo Fisher Scientific). His-DAPK2 was amplified from mouse complementary DNA and subcloned in the pET30ax vector (85761, Addgene). GST-DAPK2 expression plasmid was constructed by ligating ORF clone of DAPK2 into the *BamHI* and *EcoRI* sites of pGEX-4T-1 vector (129572, Addgene). DAPK2 (K52A), HSPA5 (S558A) and HSPA5 (S588E) were constructed by GeneArt^TM^ Site-Directed Mutagenesis System (A13282, Thermo Fisher Scientific). To generate the luciferase reporter vector bearing DAPK2 promoter region, the PCR-amplified promoters of DAPK2 from human genomic DNA was subcloned into the *SacI* and *XmaI* sites of pGL3 vector with the following primers: AGCGCCAGGATTTCAAGACTACACTGTAACCTCCAACTC (forward) and CCGGGAGTTGGAGGTTACAGTGTAGTCTTGAAATCCTGGC (reverse). Lentiviral shRNAs targeting human DAPK2 were from VectorBuilder with the oligonucleotides: AGCATAGGCGTCATCACCTACATCCTCTTAAGTGGAGCATC (sh.*Dapk2* #1) and TCACGAAATAGAAGATGGAGTTGAATTTAAGAATATTTTT

G (sh.*Dapk2* #2). The guide RNA (gRNA) with sequences CCUAUGAACUUUAUC

CAACC (#1) and UCUAAAGAGAGAUUGAGUAG (#2) for knockout of human TLR4 was annealed and cloned into the LentiGuide-Puro vector (52963, Addgene).

**Single-cell RNA sequencing (scRNA-seq) dataset analysis**

ScRNA-seq dataset (GSE167363) was downloaded from Gene Expression Omnibus (GEO). The single-cell expression matrix was processed for initial quality control, normalization, dimensional reduction and clustering using the Seurat package (version 4.1.1; https://cran.rproject.org/web/packages/Seurat/index.html). In brief, low-quality cells were excluded from the analysis when the number of features in cells < 200 or the mitochondrial unique molecular identifier (UMI) rate > 15%. We then conducted normalization according to UMI counts and mitochondrial percentages. Based on the variance/mean of all genes for integration analysis, top 2000 highly variable genes were selected in each group. The unsupervised cell clusters were generated by Louvain algorithm with multi-level refinement. Principal component analysis (PCA) was performed using the variable genes as input and statistically significant PCs were selected for tDistributed Stochastic Neighbor Embedding (tSNE) visualization. Differentially expressed genes (DEGs) between non-sepsis and sepsis non-survivor groups were identified by FindMarkers function. Benjamini-Hochberg methods was employed to calculate the false discovery rate (FDR) and cell subtypes were annotated by marker genes via applying Wilcoxon rank-sum test.

**Cycloheximide (CHX) pulse-chase experiments**

HEK293T cells were transfected with the indicated plasmids and shRNAs and incubated in 5% CO_2_ at 37 °C for 24 h. Cycloheximide was added into the media at a final concentration of 100 μg/mL. The cells were harvested at the indicated times after cycloheximide treatment. The protein turnover was analyzed by immunoblotting.

**Luciferase reporter** **assays**

HEK293T cells underwent the indicated treatments were transfected with 100 ng of pGL3 luciferase reporter plasmid bearing the promoter of DAPK2 and 1 ng of a control pRL-TK *Renilla* luciferase reporter plasmid Lipofectamine 3000 (L3000001, Invitrogen) reagent. Cells were maintained in DMEM supplemented with 10% FBS for 24 h post-transfection and then measured by the Dual-Glo Luciferase Assay System (E2940, Promega).

**Chromatin immunoprecipitation (ChIP) assay**
For ChIP assays, the LPS-stimulated THP-1 macrophages with or without BMS345541 treatment were crosslinked in 1% formaldehyde with disuccimidyl glutarate for 10 mine at room temperature and lysed on ice. After centrifugation, the nuclear lysates was subjected to sonication and then incubated with 4 µg of anti-p65 antibody overnight. Bound DNA-protein complexes were eluted by magnetic G beads and reversal of cross-links were performed at 65°C for 30 min. The purified DNA fragments were amplified by semi-quantitative PCR analyses with the following DAPK2 promoter primers: 5′-GAAGGAGTTTGAGGCCAGGA-3′ (forward) and 5′-AGATCCTCCTACCTCAGCCT-3′ (reverse).

**Immunofluorescene (IF) staining**

PMs were plated in a 24-well plate at a density of 1 × 10^5^ per well, washed thrice with PBS after LPS stimuli, fixed in cold 4% paraformaldehyde (PFA) for 15 min, permeabilized by 0.2% Triton X-100 for 10 min, blocked with 5% bovine serum albumin (BSA) for 2 h at room temperature and then incubated with the primary antibodies overnight at 4 °C. Cells were next day washed thrice with PBS containing 0.1% Tween-20 (PBST) and incubated with the fluorophore-conjugated secondary antibodies (Alexa Fluor® 488 or Alexa Fluor® 647) for 1 h at room temperature and rinsed in PBST, then mounted with mounting media containing 4’, 6-diamidino-2-phenylindole (DAPI). Cell images were acquired on a Carl Zeiss Axioimager Z1 microscope (Oberkochen, Germany).

**Supplemental Figure 1. DAPK2 is mainly upregulated in macrophages and monocytes.** Violin plot of *DAPK2* expression in different cell lineages. Data are expressed as mean ± s.d..

**Supplemental Figure 2. Macrophage DAPK2 deficiency protects against sepsis. a** PCR analysis confirming the deletion of DAPK2 in bone marrow-derived macrophages (BMDMs) of the *Dapk2^ΔMφ^* mice. **b** Immunoblotting of DAPK2 protein levels in cell lysates from bone marrow-derived macrophages (BMDMs) of *Dapk2^ΔMφ^* versus *Dapk2^fl/fl^* mice (*n* = 3 per group). **c**, **d** Bacterial load was determined in peritoneal fluid (PL, c), liver (c) and blood (d) of *Dapk2^ΔMφ^* versus *Dapk2^fl/fl^* mice with cecal ligation and puncture (CLP) operation (*n* ≥ 3 per group), respectively. n.s., no significant. **e** Representative hematoxylin and eosin (H&E) images and lung injury scores of *Dapk2^ΔMφ^* versus *Dapk2^fl/fl^* mice following LPS-induced endotoxemia (LIE) challenge (*n* ≥ 8 per group). Iso, isotype control. **f**, **g** Lung wet to dry ratio (f) and alanine aminotransferase (ALT, g) of *Dapk2^ΔMφ^* versus *Dapk2^fl/fl^* mice following LIE challenge (*n* ≥ 3 per group). Iso, isotype control; n.s., no significant. **h** Contour plots and quantification of flow cytometry with Annexin V staining in PMs of *Dapk2^ΔMφ^* versus *Dapk2^fl/fl^* mice after cecal ligation and puncture (CLP) operation (*n* = 3 per group). Iso, isotype control; n.s., no significant. **i, j** Immunfluorescence comparing caspase-3 cleavage, caspase-1 cleavage, GSDMD activation and MLKL phosphorylation in PMs of *Dapk2^ΔMφ^* versus *Dapk2^fl/fl^* mice after LPS-induced endotoxemia (LIE) challenge (i) or cecal ligation and puncture (CLP) operation (j). Scale bars: 10 μm. Data are expressed as mean ± s.d. (b-h). Two-sided Student’s t test (b-d) and Two-sided ANOVA with Bonferroni post hoc *t* test correction (e-h) was used to calculate the *P* value, respectively.

**Supplemental Figure 3. Neither mTORC1 nor macrophage phenotypic transition is indispensable for the protective effects of DAPK2 deficiency. a** Kaplan-Meier curves testing survivals of *Dapk2^ΔMφ^* versus *Dapk2^fl/fl^* mice with intraperitoneal injection of 150 µg rapamycin (Rapa) treatment after CLP operation (*n* ≥ 12 per group). **b** Kaplan-Meier curves comparing survivals of *Dapk2^ΔMφ^* mice with intraperitoneal injection of 150 µg rapamycin (Rapa) or vehicle treatment following LIE challenge (*n* ≥ 12 per group). n.s., no significant. **c** Pseudocolor plots and quantification of flow cytometry measuring M1 macrophages (CD11c^+^ F4/80^+^) in BMDMs from *Dapk2^fl/fl^* and *Dapk2^ΔMφ^* mice after LPS and IFN-γ induction (*n* = 3 per group). n.s., no significant. **d** Pseudocolor plots and quantification of flow cytometry measuring M2 macrophages (CD206^+^ F4/80^+^) in in BMDMs from *Dapk2^fl/fl^* and *Dapk2^ΔMφ^* mice after IL-4 stimuli (*n* = 3 per group). Data are expressed as mean ± s.d. (c and d). Log-rank t test (a and b) and Two-sided Student’s t test (c and d) was used to calculate the *P* value, respectively.

**Supplemental Figure 4. DAPK2 downregulates HSPA5 in a post-translantional modification-dependent manner. a** Coimmunoprecipitation assay testing the interaction of DAPK2 with HSPA5 in HEK293T cells cotransfected with Flag-tagged wild-type DAPK2 (DAPK2^Flag^) or K52A mutant DAPK2 (DAPK2 K52A^Flag^) and HA-tagged wild-type HSPA5. **b** Alignment of the evolutionarily conserved DAPK2-phosphorylating serine 588 residue in amino acid sequence of HSPA5 protein from human to african clawed frog. **c** Immunoblotting analyses comparing HSPA5 abundance in HEK293T cells transfected with Flag-tagged wild-type DAPK2 (WT) or Flag-tagged K52A mutant DAPK2 (K52A). **d** Immunoblotting analyses testing expression of HSPA5 protein in HEK293T cells with DAPK2 shRNA (sh.*Dapk2*) transfection. **e** RT-qPCR measuring HSPA5 mRNA expression in HEK293T cells transfected with Flag-tagged wild-type DAPK2 (WT) or Flag-tagged K52A mutant DAPK2 (K52A) (*n* ≥ 3 per group). n.s., no significant. **f** RT-qPCR examining HSPA5 mRNA expression in HEK293T cells with DAPK2 shRNA (sh.*Dapk2*) transfection (*n* = 3 per group). n.s., no significant. **g** Cycloheximide (CHX) pulse-chase experiments testing the turnover of HSPA5 protein in HEK293T cells with DAPK2 shRNA (sh.*Dapk2*) transfection in the presence or absence of CHX exposure for the indicated times. Data are expressed as mean ± s.d. (e and f). Two-sided ANOVA with Bonferroni post hoc *t* test correction (e and f) was used to calculate the *P* value.

**Supplemental Figure 5. HSPA5-IRE1α axis contributes to the detrimental role of macrophage DAPK2 in sepsis. a** Contour plots and quantification of flow cytometry with Annexin V staining in PMs of cecal ligation and puncture (CLP)-operated *Dapk2^ΔMφ^* mice receiving vehicle, YUM70 treatment and YUM70 plus KIRA6 cotreatment (*n* = 3 per group). **b** Representative hematoxylin and eosin (H&E) images and lung injury scores of *Dapk2^ΔMφ^* mice receiving vehicle, YUM70 treatment and YUM70 plus KIRA6 cotreatment after LIE challenge (*n* ≥ 9 per group). Data are expressed as mean ± s.d. (a and b). Two-sided ANOVA with Bonferroni post hoc *t* test correction (a and b) was used to calculate the *P* value.

**Supplemental Figure 1**


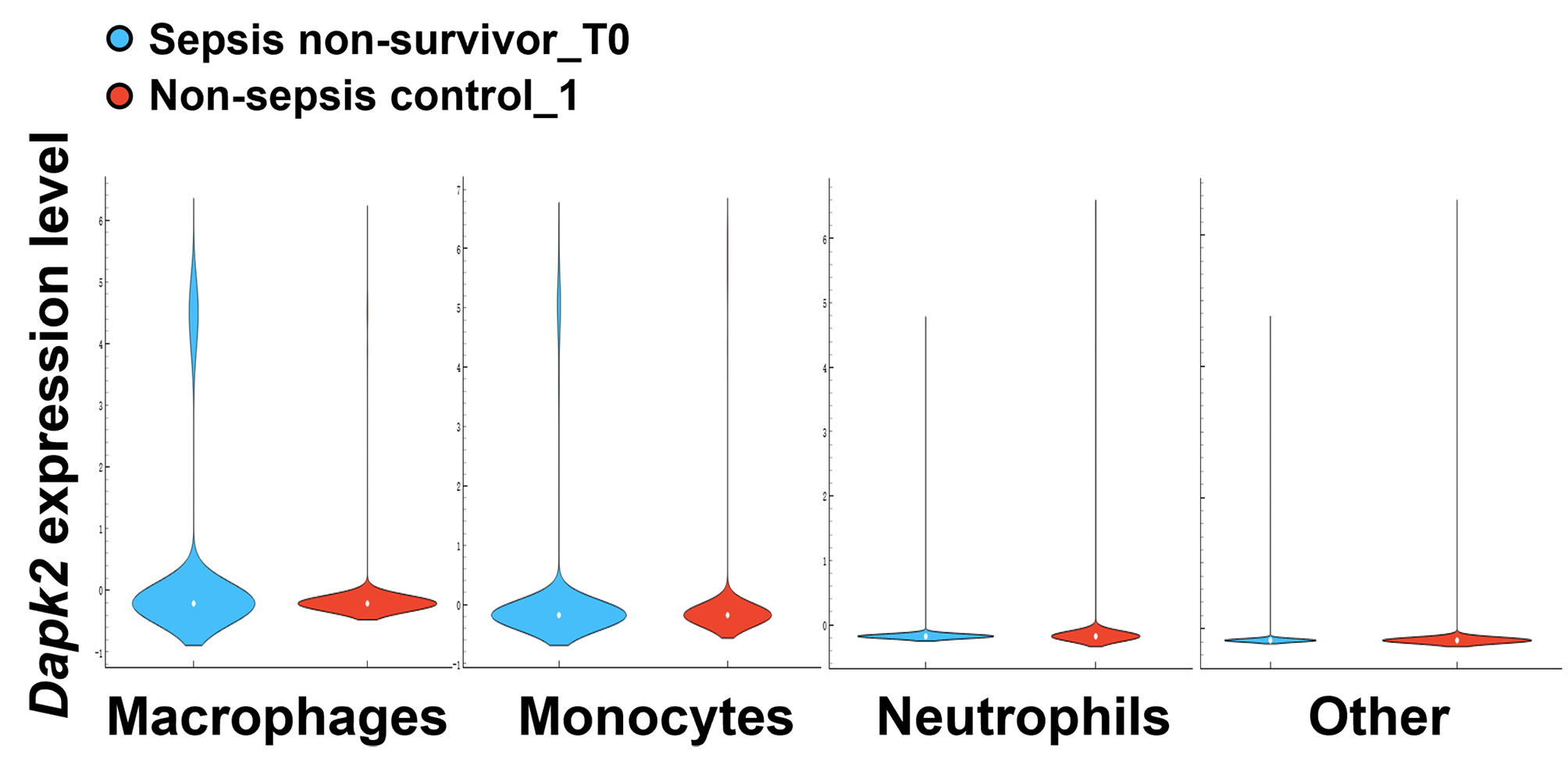


**Supplemental Figure 2**


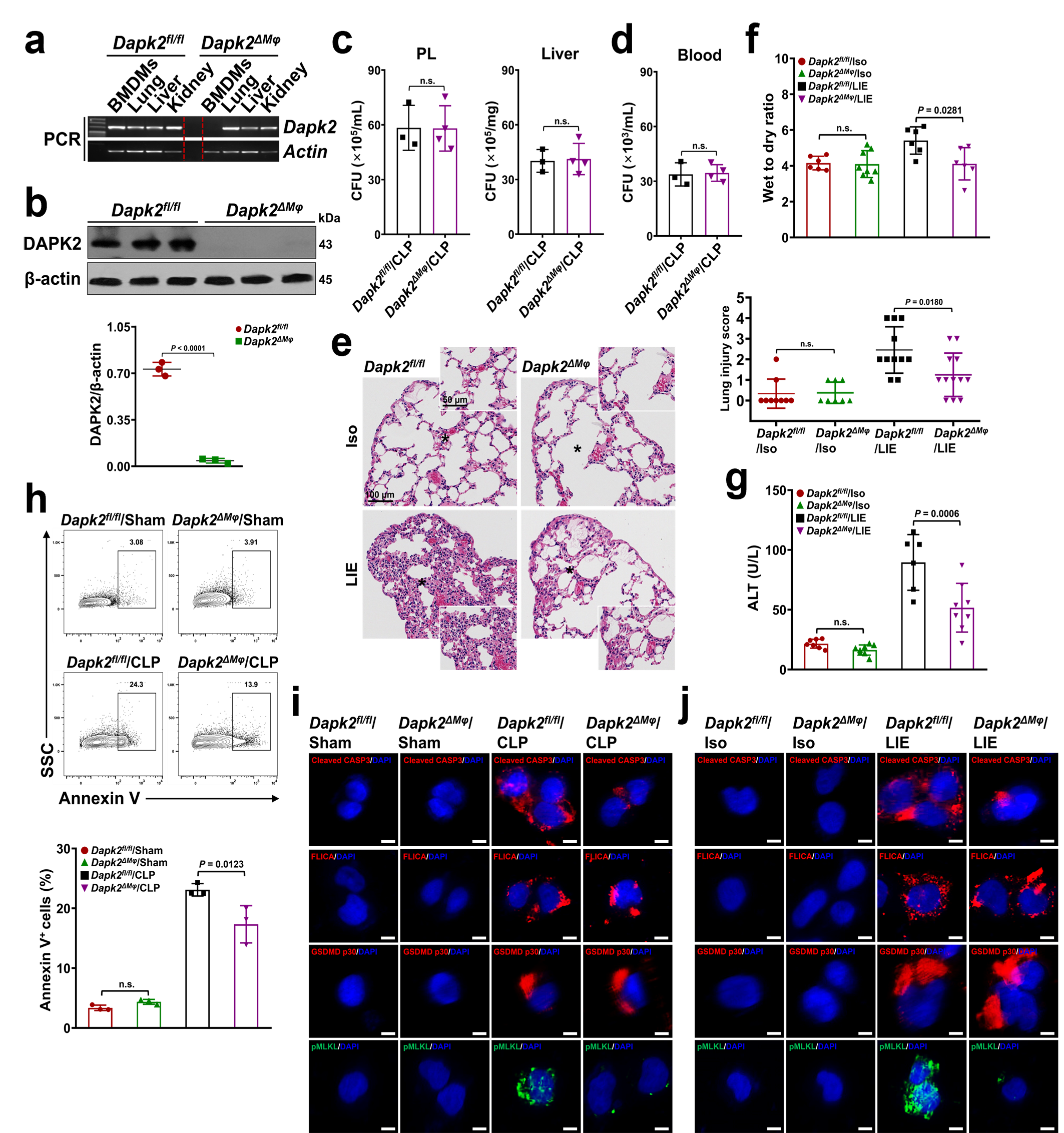


**Supplemental Figure 3**


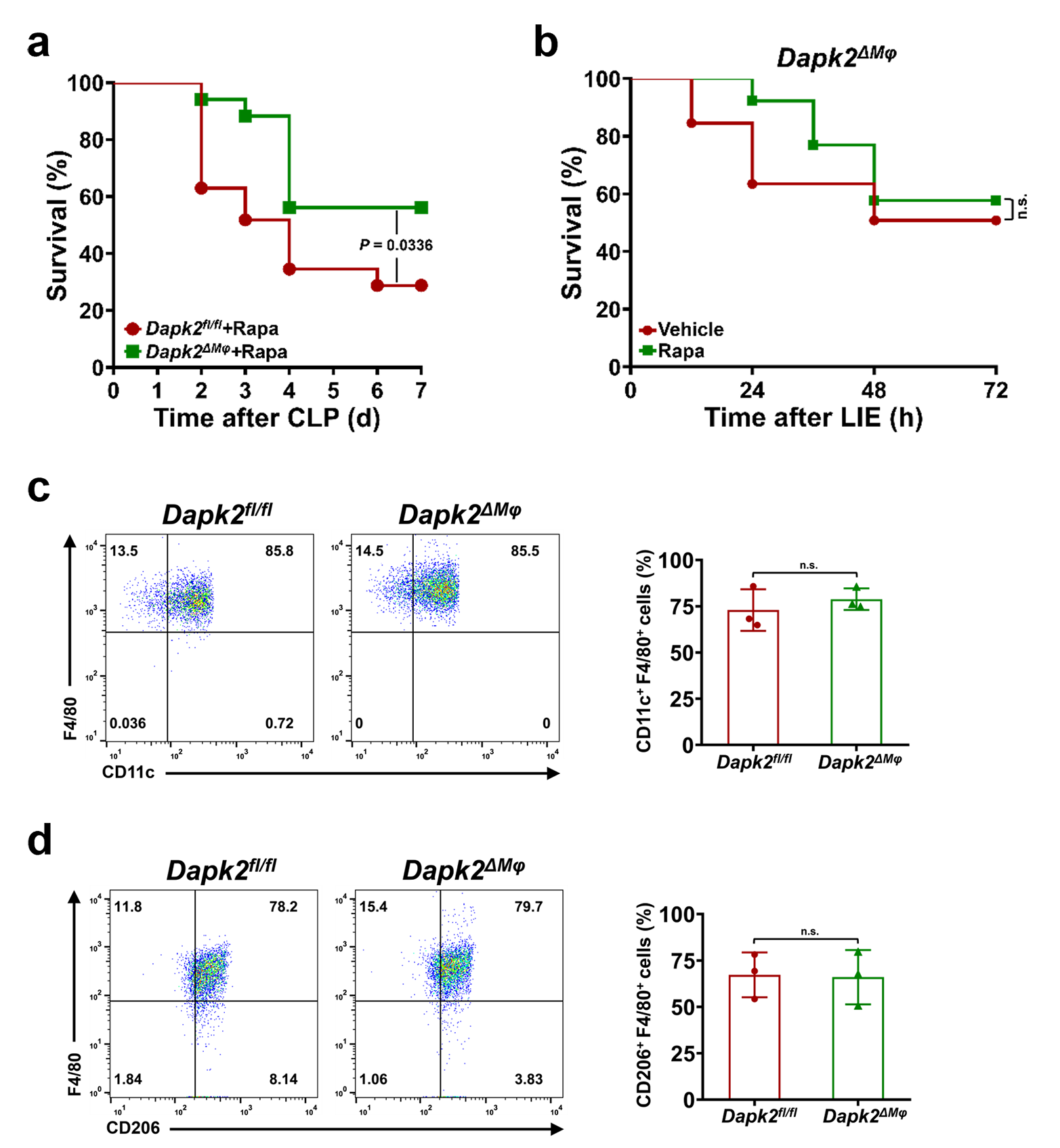


**Supplemental Figure 4**


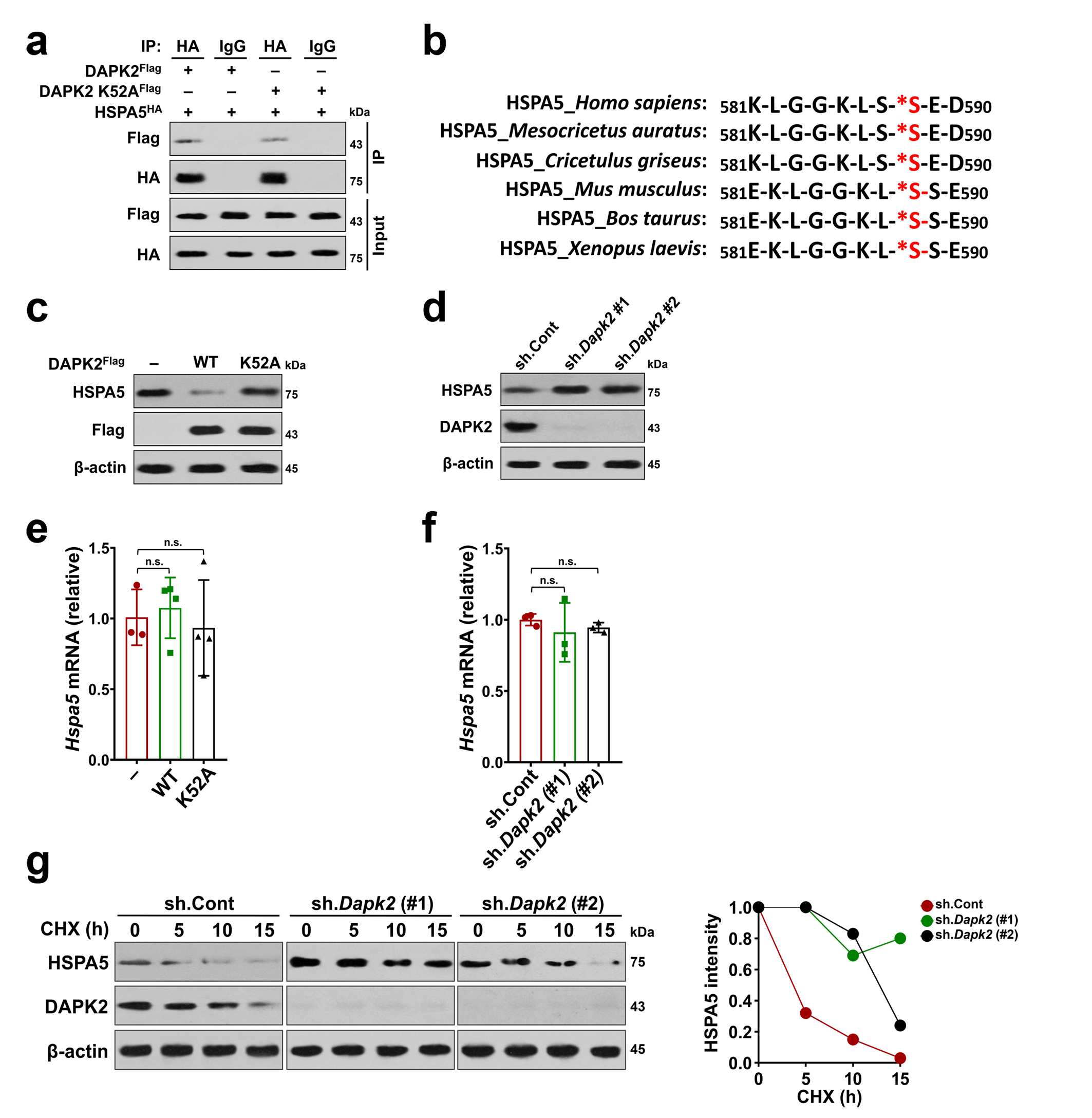


**Supplemental Figure 5**


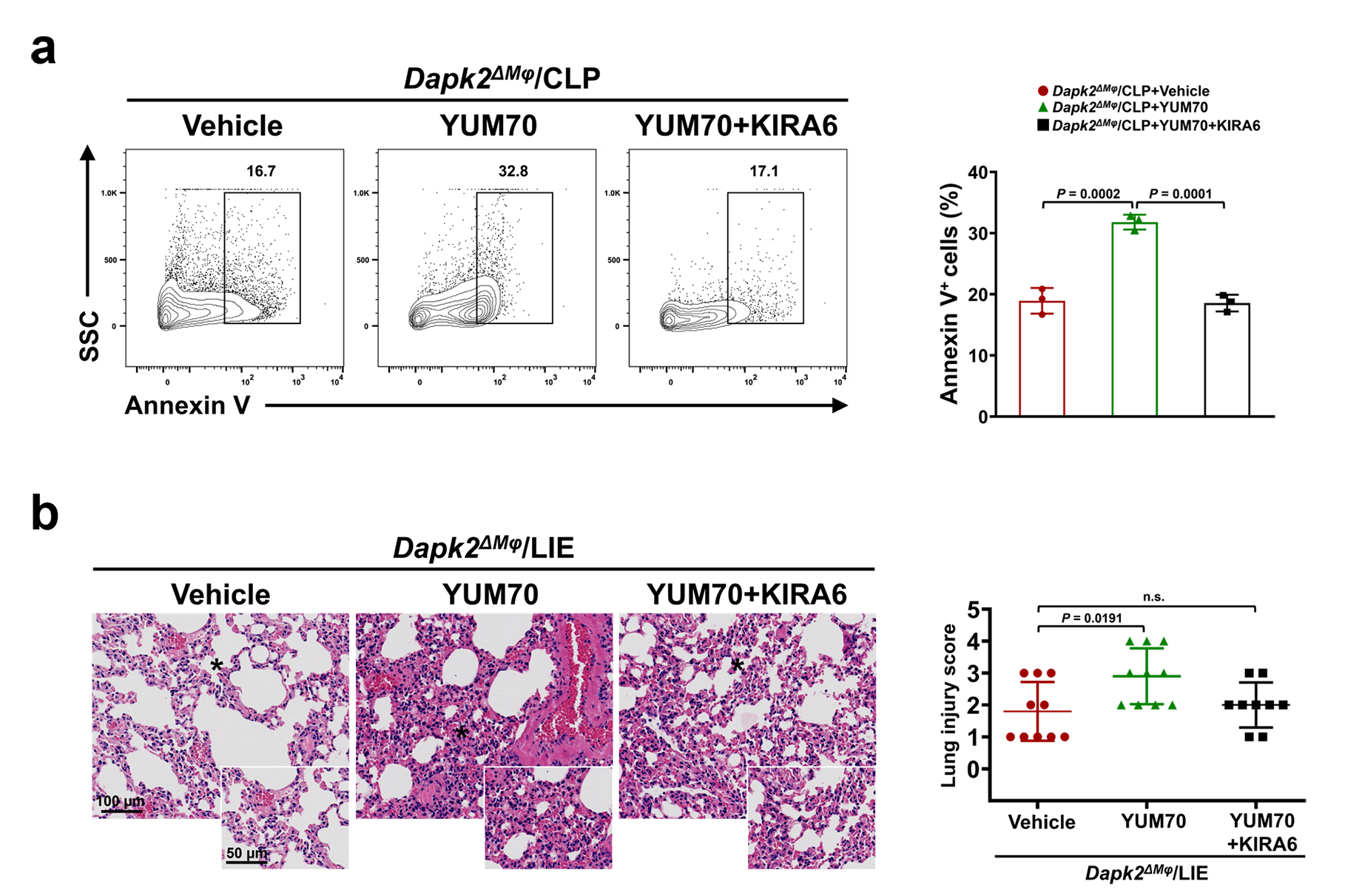

Supplement: Supplementary file 1 — Supplementary Material 1. [file 43556_2026_501_MOESM1_ESM.doc]
